# Structural brain correlates of serum and epigenetic markers of inflammation in major depressive disorder

**DOI:** 10.1016/j.bbi.2020.11.024

**Published:** 2021-02

**Authors:** Claire Green, Xueyi Shen, Anna J. Stevenson, Eleanor L.S. Conole, Mathew A. Harris, Miruna C. Barbu, Emma L. Hawkins, Mark J. Adams, Robert F. Hillary, Stephen M. Lawrie, Kathryn L. Evans, Rosie M. Walker, Stewart W. Morris, David J. Porteous, Joanna M. Wardlaw, J Douglas Steele, Gordon D. Waiter, Anca-Larisa Sandu, Archie Campbell, Riccardo E. Marioni, Simon R. Cox, Jonathan Cavanagh, Andrew M. McIntosh, Heather C. Whalley

**Affiliations:** a Division of Psychiatry, University of Edinburgh, Edinburgh, UK; b Centre for Genomic and Experimental Medicine, Institute of Genetics and Molecular Medicine, University of Edinburgh, Edinburgh, UK; c UK Dementia Research Institute, Edinburgh Medical School, University of Edinburgh, Edinburgh, UK; d Lothian Birth Cohorts Group, University of Edinburgh, Edinburgh, UK; e Centre for Cognitive Ageing and Cognitive Epidemiology, Department of Psychology, University of Edinburgh, Edinburgh, UK; f Centre for Clinical Brain Sciences, University of Edinburgh, Edinburgh, UK; g Division of Imaging Science and Technology, School of Medicine, University of Dundee, Dundee, UK; h Aberdeen Biomedical Imaging Centre, Institute of Medical Sciences, University of Aberdeen, Aberdeen, UK; i Institute of Infection, Immunity & Inflammation, College of Medical and Veterinary Life Sciences, University of Glasgow, Glasgow, UK; j Institute of Health and Wellbeing, College of Medical and Veterinary Life Sciences, University of Glasgow, Glasgow, UK

**Keywords:** Major depressive disorder, Depression, Inflammation, C-reactive protein, CRP, Methylation, Brain morphology, Brain structure, White matter integrity, MRI

## Abstract

Inflammatory processes are implicated in the aetiology of Major Depressive Disorder (MDD); however, the relationship between peripheral inflammation, brain structure and depression remains unclear, partly due to complexities around the use of acute/phasic inflammatory biomarkers.

Here, we report the first large-scale study of both serological and methylomic signatures of CRP (considered to represent acute and chronic measures of inflammation respectively) and their associations with depression status/symptoms, and structural neuroimaging phenotypes (T1 and diffusion MRI) in a large community-based sample (Generation Scotland; N_MDD cases_ = 271, N_controls_ = 609).

Serum CRP was associated with overall MDD severity, and specifically with current somatic symptoms- general interest (β = 0.145, P_FDR_ = 6 × 10^−4^) and energy levels (β = 0.101, P_FDR_ = 0.027), along with reduced entorhinal cortex thickness (β = −0.095, P_FDR_ = 0.037). DNAm CRP was significantly associated with reduced global grey matter/cortical volume and widespread reductions in integrity of 16/24 white matter tracts (with greatest regional effects in the external and internal capsules, β_FA_= −0.12 to −0.14). In general, the methylation-based measures showed stronger associations with imaging metrics than serum-based CRP measures (βaverage = −0.15 versus βaverage = 0.01 respectively).

These findings provide evidence for central effects of peripheral inflammation from both serological and epigenetic markers of inflammation, including in brain regions previously implicated in depression. This suggests that these imaging measures may be involved in the relationship between peripheral inflammation and somatic/depressive symptoms. Notably, greater effects on brain morphology were seen for methylation-based rather than serum-based measures of inflammation, indicating the importance of such measures for future studies.

## Highlights

• First study of both serum and DNAm CRP associations with depression/neuroimaging.
• Serum CRP is associated with somatic symptoms and reduced entorhinal cortex thickness.
• DNAm CRP is associated with widespread reductions in white matter integrity.
• Evidence for central effects of peripheral inflammation from serum and DNAm markers.

## 1 Introduction

Major depressive disorder (MDD) is the most common mental health condition in the general population (Whiteford et al., 2013). It is a heritable disorder (Howard et al., 2019, Sullivan et al., 2000) that can be linked to a diminished functioning and quality of life, medical morbidity, and mortality (Marcus et al., 2012, Sinyor et al., 2016). Activation of the peripheral immune system has been consistently associated with MDD and is implicated in its pathogenesis (Bhattacharya et al., 2016, Dantzer et al., 2008, Haapakoski et al., 2015, Raison et al., 2013, Strawbridge et al., 2015). Evidence has shown that markers of inflammation are upregulated in peripheral and central nervous system tissue of individuals with depression compared to healthy controls, including increased concentrations of proinflammatory cytokines and immune mediators in cerebrospinal fluid (Dowlati et al., 2010, Lindqvist et al., 2009, Liu et al., 2012, Pandey et al., 2012). Furthermore, a meta-analysis of randomised control trials (n = 2370) of proinflammatory cytokine inhibitors showed these treatments significantly improved depressive symptoms compared with placebo, indicating a potentially casual role of inflammation in MDD (Kappelmann et al., 2018).

One of the most common approaches to assess peripheral inflammation is by measuring serum levels of C-reactive protein (CRP). CRP plays a key role in human inflammation and can provide a proxy estimate for inflammatory activity (Lowe, 2005). It is widely used in clinical practice as a marker of acute inflammation and is a candidate biomarker for investigating inflammatory processes in MDD (Osimo et al., 2019). Meta-analyses have shown that serum CRP levels are reliably elevated in MDD (Valkanova et al., 2013) and have been found to predict future development of depression as well as resistance to standard antidepressant therapies (Au et al., 2015, Chamberlain et al., 2019, Strawbridge et al., 2015).

One hypothesis is that peripheral inflammation contributes to depressive symptoms through effects on the brain. There is increasing evidence that peripheral inflammation has effects on the brain via humoral and neural routes subsequently impacting upon neural cell function, changes in functional/structural connectivity and behavioural changes including depressive symptoms (Brydon et al., 2008, Harrison et al., 2016, Harrison et al., 2009, Savitz et al., 2015a, Savitz et al., 2015b, Savitz et al., 2013). However, at present there is little evidence of associations between elevated serum CRP in MDD and alterations in brain structure or morphology. One exception is the large imaging study by Opel and colleagues that reported significantly increased CRP levels in association with reduced grey matter volume in 514 patients with MDD compared to 359 healthy controls, however associations with white matter integrity were not investigated (Opel et al., 2019). Further, although serum CRP is viewed as a proxy measure of inflammatory activity, it can be influenced by a number of current state factors such as recent infections, injuries, body mass index (BMI), or chronic inflammatory conditions (Kathiresan et al., 2006). Cross-sectional serum measures may also not capture chronic low grade/sub-acute inflammation over time, which is considered important in MDD (Goldsmith et al., 2016, Haapakoski et al., 2015).

We therefore investigated a methylation marker of chronic inflammation using a recent large epigenome wide association study (EWAS) of CRP (Ligthart et al., 2016). From the CpG sites identified, a methylation score for CRP (DNAm CRP) was created (Barker et al., 2018); the score has since been generated in participants of Generation Scotland and showed greater longitudinal stability compared to serological CRP (n = 7028) (Stevenson et al., 2020). Since this measure of inflammation may be less prone to the acute effects of cross-sectional measures of serum CRP described above, DNAm CRP may provide a more stable signature of chronic inflammatory states compared to cross-sectional serum CRP (Byun et al., 2012, Talens et al., 2010). Indeed, methylation risk scores of other phenotypes including MDD itself have demonstrated discriminatory utility (Barbu et al., 2020).

Here we present findings from both serological measures of CRP and a novel methylomic approach to investigate the role of inflammation in MDD with a comprehensive range of structural brain phenotypes, including white matter microstructure (n = 189 phenotypes). The current study examines a large community-based sample (n = 880) to investigate (i) associations between serum CRP and DNAm CRP and MDD symptoms, (ii) associations between both CRP (serum CRP and DNAm CRP) measures and structural imaging phenotypes (TI and diffusion MRI) and (iii) interaction effects between both measures of CRP and MDD diagnosis to determine the differential relationship of these inflammatory markers and imaging associations in depression.

## 2 Methods and materials

### 2.1 Participants

The participants in this study were recruited as part of the ‘STratifying Resilience and Depression Longitudinally’ (STRADL) study (2015–2019) which re-contacted participants from the Generation Scotland: Scottish Family Health Study (GS) via post for further assessment of mental health, specifically depression. Full details of the STRADL cohort and GS protocol are published elsewhere (Habota et al., 2019, Navrady et al., 2018, Romaniuk et al., 2019, Rupprechter et al., 2020, Smith et al., 2013, Stolicyn et al., 2020). A total of 880 unrelated participants were included in this study, 880 individuals had serum CRP and symptoms data, 796 individuals had both serum CRP and T1 neuroimaging data, and of these 764 individuals also had diffusion MRI (DTI) data. In terms of DNAm CRP, 598 individuals had DNAm CRP and symptom data, 590 individuals had both DNAm CRP and T1 data and of these 565 also had DTI data (Table 1).

**Table 1:** Participant Demographics.

	Variable	Unit	Total (Cases and Controls)	Controls	MDD Cases	*p*-value
Demographics*	Age^a^	Years (M, SD)	59.7 (9.6)	60.8 (9.3)	57.3 (9.7)	<0.01
	Sex^b^	Females (n, %)	504 (57.3)	313 (51.4)	191 (70.5)	<0.01
	BMI^a^	Mean (SD)	28.2 (5.9)	27.6 (5.5)	29.6 (6.5)	<0.01
	SCID Diagnosis	n	880	609	271	–
	Total QIDS score^a^	Mean (SD)	4.7 (3.7)	3.6 (2.3)	7.1 (5)	<0.01
	Serum CRP status^b^	<4mg/L (n)	714	507	207	0.02
		≥4 mg/L (n)	166	102	64	
	DNAm CRP score^a^	Mean (SD)	−0.012(0.0008)	−0.012(0.0008)	−0.0119(0.0007)	<0.01

n/analyses	Serum CRP and Symptoms	n	880	609	271	–
	Serum CRP and T1	n	796	557	239	–
	Serum CRP and DTI	n	764	537	227	–
	DNAm CRP and Symptoms	n	598	402	196	–
	DNAm CRP and T1	n	590	397	193	–
	DNAm CRP and DTI	n	565	382	183	–

*Demographics calculated on 880 participants with serum CRP and symptoms data, a = Wilcoxon *t*-test, b = Chi squared test.

Ethical approval for STRADL was formally obtained from the NHS Tayside committee on research (reference 14/SS/0039), and all participants provided their written informed consent.

### 2.2 Clinical assessment

A full medical history was obtained and updated from previous GS baseline assessment (Smith et al., 2013) and any new diagnoses or medical episodes recorded at the imaging assessment. Health and lifestyle data were also collected, as were physical measurements such as height and weight, from which BMI was derived as a covariate of interest (Habota et al., 2019). Smoking status was collected from GS baseline assessments as were the number of smoking pack years- full details have been reported by Barbu and colleagues (Barbu et al., 2020).

Participants in STRADL completed a broad range of tests designed to assess depression incidence/severity. All participants were screened for a lifetime history of MDD. A research version of the Structured Clinical Interview for DSM disorders (SCID) (First et al., 2002) was used to assess symptoms of mood disorder. Diagnostic criteria were based on the Diagnostic and Statistical Manual of Mental Disorders (DSM-IV-TR). From this, participants were given a binary score of no history of depression (0) or lifetime episode of depression (1). Using this definition our study had 271 cases of lifetime depression and 609 controls (Table 1). No participants met criteria for lifetime bipolar disorder. The Quick Inventory of Depressive Symptomatology (QIDS) (John Rush et al., 1986), a 16-item questionnaire, was employed in order to assess the severity of current depressive symptoms at the time of assessment among study participants. From this, a total QIDS score was calculated as a measure of current depression severity for analyses purposes. Additional analyses looking at further clinical features of MDD and associations with serum CRP and DNAm CRP were also conducted and are provided in supplementary materials (Table S23).

### 2.3 C-reactive protein measurement (CRP)

To obtain serum CRP levels, venepuncture was employed using a butterfly needle kit. Blood was extracted into clot activator gel for serum separation. CRP samples were taken and sent to NHS laboratories (Ninewells Hospital/Aberdeen Royal Infirmary) for analysis. The low-sensitivity assay utilized in CRP analyses possessed a detection threshold of 4 mg/L and CRP levels below the 4 mg/L detection threshold were recorded as 0. For analyses purposes, CRP levels were stratified based on this threshold limit into clinically relevant groups: <4 mg/L (clinically normal) and ≥ 4 mg/L (clinically elevated) (Ridker, 2003). Blood samples were taken concurrently with imaging analyses and depression measures.

### 2.4 DNAm CRP score calculation

Blood samples used to generate the DNAm CRP score were collected at the Generation Scotland baseline appointment (between 2006 and 2011) and DNA methylation was profiled using the Illumina Human-MethylationEPIC BeadChip in two different sets. Pre-processing and quality control steps for both sets of methylation data have previously been fully reported (Madden et al., 2020, Stevenson et al., 2020).

Full details of the calculation of the DNAm CRP score in GS have been reported previously (Stevenson et al., 2020). Briefly, methylation beta values were extracted for 6 CpG sites shown to have the strongest evidence of a functional association with serum CRP levels as shown by Lighthart and colleagues (n = 8863 and 4111 of European and African ancestries, respectively) (Ligthart et al., 2016). One of the 7 CpG sites (cg06126421) in the original study was unavailable in the GS dataset and was therefore not included in the current analysis resulting in 6 CpG sites. The beta values for the six CpG sites associated with serum CRP were then multiplied by their respective regression weights and summed to generate a single score for each STRADL participant (Stevenson et al., 2020). As all the EWAS regression weights were negative, a higher DNAm CRP score corresponds to a score closer to zero.

### 2.5 MRI acquisition and analyses

STRADL participants were scanned at two centres: the Ninewells Hospital in Dundee and at the Aberdeen Royal Infirmary in Aberdeen. Participants in Dundee were scanned using a Siemens 3T Prisma-FIT (Siemens Healthineers, Erlangen, Germany) with a 20-channel head and neck coil and a back-facing mirror (software version VE11, gradient with max amplitude 80 mT/m and maximum slew rate 200 T/m/s). In Aberdeen, participants were imaged on a 3 T Philips Achieva TX series MRI system (Philips Healthcare, Best, Netherlands) with a 32-channel phased-array head coil with a back-facing mirror (software version 5.1.7; gradients with maximum amplitude 80 mT/m and maximum slew rate 100. Both study centres followed the same protocol including structural sequences (Habota et al., 2019). 3T MRI scans were anonymised at the time of acquisition and only the T1 and DTI data are utilized in this study. Scanning site was included as a covariate in statistical analyses. Full details of the imaging sequences and parameters are provided in supplementary materials.

T1 structural measures were processed using FreeSurfer version 5.3 (Dale et al., 2002) to quantify the volumes of 14 subcortical structures as well as the volumes, surface area and thickness of 34 cortical regions per hemisphere according to the Desikan-Killany atlas (Desikan et al., 2006). Full details of the Freesurfer Quality Control (QC) steps are provided in supplementary materials and have also been reported in full previously (Neilson et al., 2019). Measures of thickness, surface area and volume were derived for each of the 68 cortical regions. The volumes of 14 subcortical structures – left and right accumbens area, amygdala, caudate nucleus, hippocampus, pallidum, putamen and thalamus – were also extracted from FreeSurfer output. Global measures of cortical volume, surface area and thickness were also derived, as well as 5 summed lobar measures (frontal, parietal, temporal, occipital and cingulate; Table S1). The number of QC edits made per individual were recorded to use as a covariate in statistical analyses.

For DTI data, pre-processing and quality control was performed using standard tools available from FSL (https://fsl.fmrib.ox.ac.uk/fsl/fslwiki). Tract Based Spatial Statistics (TBSS) was carried out according to the ‘The Enhancing NeuroImaging Genetics through Meta-Analysis’ (ENIGMA) Consortium DTI protocol (http://enigma.ini.usc.edu/protocols/dti-protocols/). Region of interest (ROI) extraction analyses were then performed also using ENIGMA protocols to extract fractional anisotropy (FA) and mean diffusivity (MD) measures (http://enigma.ini.usc.edu/protocols/dti-protocols/). White matter tracts were categorised using the Johns-Hopkins University DTI-based white matter atlas (Mori et al., 2007). This resulted in 5 unilateral tracts and 19 bilateral tracts, as well as an average measure, for FA and MD. This included ten association fibres, three commissural fibres, eight projection fibres and four thalamic radiations (Table S1).

### 2.6 Statistical analyses

#### 2.6.1 Subcortical/cortical measures

For all cortical and subcortical measures, age, sex, BMI, imaging batch, number of image edits per individual, hemisphere, assessment centre and standardised intracranial volume (ICV) were set as covariates in mixed-effect linear models for both serum CRP and DNAm CRP analyses. Additionally, smoking status and pack years smoked were included in all DNAm CRP analyses given the effects of smoking on a range of methylation-based measures (Joehanes et al., 2016). For unilateral structures and global/lobar measures, a general linear model was applied as above. Hemisphere was controlled for as a within-subject variable in all bilateral structural neuroimaging phenotypes, using mixed-effect models.

#### 2.6.2 White matter integrity

For DTI measures, age, sex, BMI and assessment centre were included as covariates for all analyses and methylation set/smoking variables were additionally included for DNAm CRP analyses. Global integrity was determined by applying principal component analysis (PCA) on the 24 tracts to extract a latent measure. Scores of the first unrotated principal component of FA/MD were extracted and set as the dependent variable (proportion of variance explained by the first principal component is provided in Table S2). We then separately examined four subsets of white matter tract for which scores of the first unrotated principal component were also extracted: (a) association fibres, (b) commissural fibres, (c) projection fibres and (d) thalamic radiations. The tracts included in the four subsets are provided in supplementary materials (Table S1). Finally, we examined each white matter tract individually. Mixed-effect linear models were used for the measures of bilateral white matter tracts correcting for hemisphere as a within-subject variable, consistent with above, while general linear models were used for the unilateral midline tracts.

#### 2.6.3 Statistical models

All analyses were conducted using R (version 3.2.3) in a Linux environment. As GS is a family-based study we randomly included one participant per family in order to have an unrelated sample. Randomisation was conducted in R using the ‘rnorm’ function to create a random seed variable for each participant and one individual per family with the highest random number was included in subsequent analyses, excluding all other family members. Linear mixed-effects models (function ‘lme’ in R package ‘nlme’) and general linear models (function ‘glm’ in R package ‘stats’) were used to investigate structural brain metrics (Shen et al., 2020, Shen et al., 2017). False Discovery Rate (FDR) multiple comparison correction was applied to all bilateral/unilateral structures, lobes and white matter tracts, referred to as P_FDR_ in this report, using the ‘p.adjust’ function in R and all betas were standardised. FDR correction was also applied over each sub-analysis. We investigated: (i) associations between serum CRP and DNAm CRP and depression symptoms, (ii) associations between both CRP measures and structural imaging phenotypes (T1 and DTI) and (iii) CRP × MDD interaction effects using both measures of CRP (serum and DNAm), and case/control MDD status. Further analyses controlling for concurrent smoking in serum CRP-MDD associations (Table S22) and controlling for days between methylation and serological/MRI appointments in DNAm CRP associations are also provided in supplementary materials (Supplementary Tables S24-28).

## 3 Results

### 3.1 Demographics

Descriptive statistics of the key variables as well as sample numbers for each analysis in this study are presented in Table 1. Case-control MDD and QIDS associations on the structural imaging metrics across the full sample are also provided in supplementary materials (Tables S16–S21).

### 3.2 Associations between serum CRP and DNAm CRP with depression symptoms

#### 3.2.1 Serum C-reactive protein

**Fig. 1:**
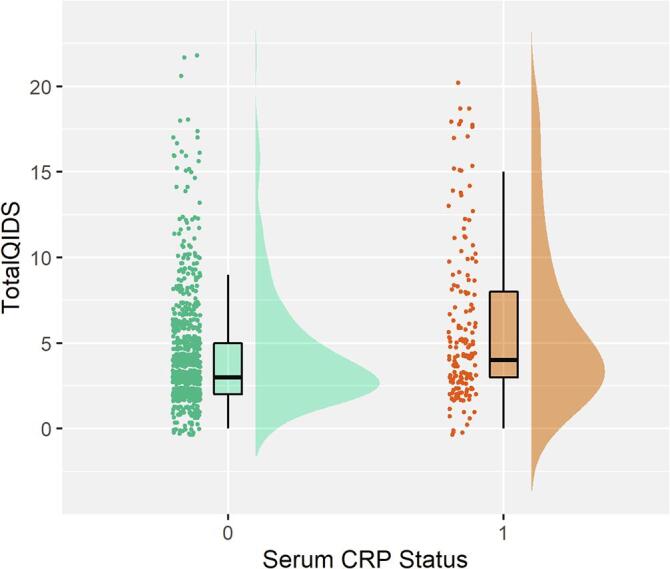
Raincloud plot of Serum CRP status associations with total QIDS scores. Serum CRP status 0 represents individuals with serum CRP < 4 mg/L and serum CRP status of 1 represents individuals with a serum CRP level of 4 mg/L or higher.

There were no significant associations between serum CRP and case/control MDD status. However, increased serum CRP levels were significantly associated with increased depressive symptoms as measured by the total QIDS score (β = 0.073, P_FDR_ = 0.033; Fig. 1). For the separate QIDS items, increased serum CRP levels were significantly associated with decreased energy levels (β = 0.101, P_FDR_ = 0.027) and decreased general interest (β = 0.145, P_FDR_ = 6 × 10^−4^).

A further supplementary analysis controlling for smoking status in serum CRP associations with depression is presented in supplementary materials (Table S22). This sample had reduced power of 101 participants, however, effect sizes for the main findings remained similar, total QIDS (β = 0.06, P_FDR_ = 0.07), with general interest remaining FDR significant after additional correction for smoking status (β = 0.1, P_FDR_ = 0.02).

#### 3.2.2 DNAm CRP

There were no significant associations between the DNAm CRP score and MDD case/control status, or any of the measures of depression symptoms from the QIDS. A supplementary analysis looking at further clinical features of MDD (age of onset, recurrence, QIDS severity) and associations with serum CRP and DNAm CRP were also null (Table S23).

### 3.3 Associations of serum C-reactive protein and DNAm CRP and structural brain metrics

#### 3.3.1 Serum C-reactive protein

**Fig. 2:**
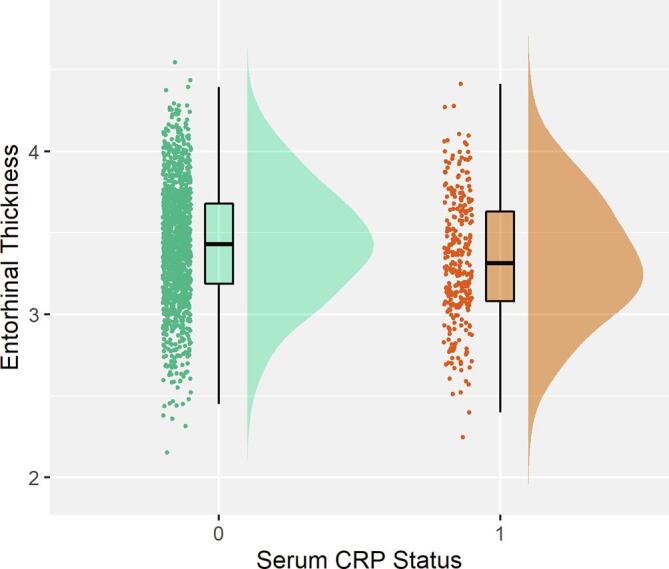
Raincloud plot of Serum CRP status associations with entorhinal thickness. Serum CRP status 0 represents individuals with serum CRP < 4 mg/L and serum CRP status of 1 represents individuals with a serum CRP level of 4 mg/L or higher.

**Fig. 3:**
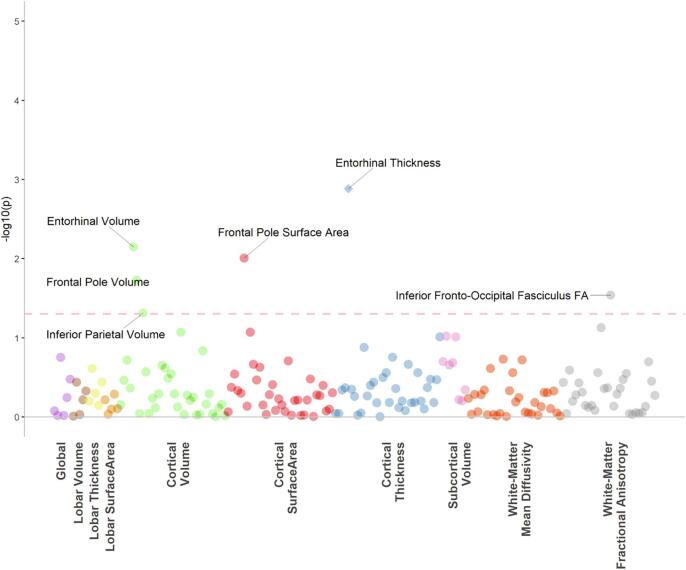
The dotted line indicates the p value threshold 0.05. Each dot represents one structural brain phenotype. Each colour represents one imaging modality. The diamonds represent phenotypes that are also significant after FDR correction.

There were no significant associations between serum CRP status and any global or lobar brain summary measures. For individual regions however, we found elevated CRP status was significantly associated with reduced thickness of the entorhinal cortex (β = −0.095, P_FDR_ = 0.037; Fig. 2). There were no significant associations between serum CRP status on any of the DTI measures, globally or regionally (Fig. 3).

#### 3.3.2 DNAm CRP

**Fig. 4:**
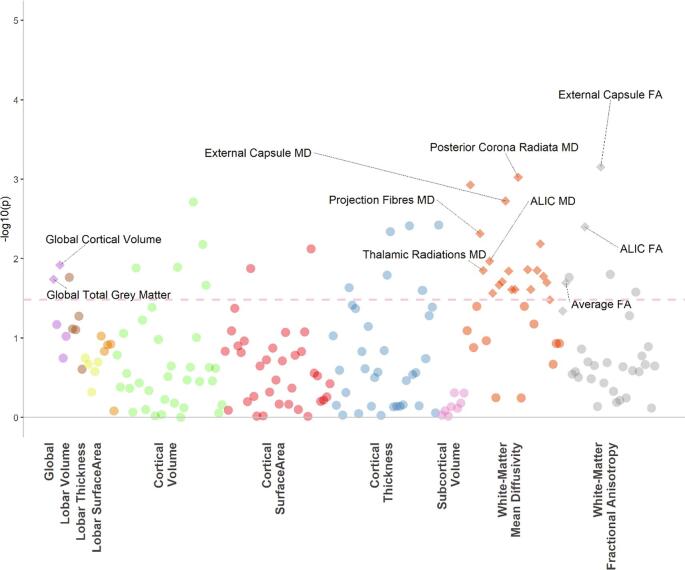
The dotted line indicates the p value threshold 0.05. Each dot represents one structural brain phenotype. Each colour represents one imaging modality. The diamonds represent phenotypes that are also significant after FDR correction.

For DNAm CRP (n = 590), we report significant associations between an increased DNAm CRP score and smaller global grey matter (β = −0.06, p = 0.02) and smaller global cortical volume (β = −0.1, p = 0.01; Fig. 4). There were no FDR significant associations with any of the regional structural measures.

With regard to DTI measures (n = 565), increased DNAm CRP scores were significantly associated with decreases in white matter microstructure, including negative associations with gFA (β = −0.07, p = 0.04), FA in the external capsule (β = −0.14, P_FDR_ = 0.02) and the anterior limb of the internal capsule (ALIC; β = −0.12, P_FDR_ = 0.048).

There were also significant associations with MD values for projection fibres (β = 0.098, P_FDR_ = 0.019) and thalamic radiations (β = 0.079, P_FDR_ = 0.028). We also tested the effects of the DNAm CRP score for MD of individual tracts and found FDR significant effects for 16/24 white matter tracts (Fig. 4; Table S9). One of the largest effect sizes found for an individual tract was for the ALIC (β = 0.1, P_FDR_ = 0.042).

Results for an additional analysis controlling for the time interval between methylation data collection and imaging/serological collection (Tables S24–28) were largely similar to the above findings. Results remained the same for all global and FA findings, and 14/16 of the previous MD tract findings remained FDR significant, with the largest effect sizes in the same tracts (the corticospinal tract and inferior-fronto-occipital fasciculus were no longer FDR significant, both P_FDR_ = 0.056).

### 3.4 Interaction effects between peripheral inflammation and depress on structural brain metrics

Lastly, we tested interaction effects between both peripheral markers of inflammation and case/control status and structural brain measures. There were no significant interaction effects between serum CRP or DNAm CRP with MDD case/control status on any of the brain metrics investigated (Tables S10-S15).

## 4 Discussion

Using data from a large community-based sample, we report associations between serological and methylomic markers of CRP with 189 structural neuroimaging phenotypes and their interaction effects with MDD diagnosis. We show that serological CRP exhibited significant associations with overall current MDD symptoms, in particular somatic symptoms, as measured by QIDS scores. In terms of brain morphology, serum CRP was associated with decreased thickness of the entorhinal cortex, whereas DNAm CRP was more robustly associated with widespread imaging features including decreased global cortical volume, grey matter and decreased white matter integrity, with the greatest loss of integrity in the external capsule and the ALIC (as indexed by decreased FA and increased MD). Neither serum CRP or DNAm CRP had significant interaction effects with MDD case/control status with brain structure, indicating these relationships are not specific to the presence of a formal lifetime clinical diagnosis.

We found that increased serum CRP levels were associated with increased depressive symptoms, particularly two somatic symptoms, general interest and energy levels, which is consistent with the existing literature. Previous studies of inflammation and depressive symptoms have also found that inflammatory markers are associated with somatic/neurovegetative symptoms of depression including fatigue, impaired sleep and activity, rather than psychological or cognitive symptoms (Chu et al., 2019, Duivis et al., 2013, Iob et al., 2020, Jokela et al., 2016, Köhler-Forsberg et al., 2017, White et al., 2017). These findings suggest that elevated serum CRP may be a reliable biomarker of somatic symptoms and provides further support for ‘sickness behaviour’ theories of depression (Dantzer et al., 2008) where such features are thought to stem from inflammatory responses (Iob et al., 2020). This is relevant in the mechanistic treatment of MDD, whereby somatic symptoms could potentially be alleviated by therapeutics that target the immune system/pro-inflammatory mechanisms.

Across the whole sample, we also report that higher serum CRP was associated with a thinner entorhinal cortex. This region has also been associated with CRP in previous research. A study by Bettcher and colleagues found CRP was associated with smaller left medial temporal lobe volumes, which included the entorhinal cortex, and found that those with detectable levels of CRP demonstrated poorer performance in cognitive tasks (Bettcher et al., 2012). This area has also been associated with the development of somatic symptoms in previous research (Wei et al., 2015) where the medial temporal lobe is thought to be involved in the emotional component of somatic complaints, consistent with our findings above (Phelps, 2006). Previous research on the effects of inflammation on the entorhinal cortex are primarily pre-clinical or in the context of Alzheimer’s disease (AD); however, chronic neuroinflammation is associated with neuronal loss and BBB leakage in this region and the entorhinal cortex may represent an area that is particularly vulnerable to the effects of inflammation (Hauss-Wegrzyniak et al., 2002, Montagne et al., 2020). The current findings therefore also indicate the potential importance of this region in the aetiology of somatic symptoms in MDD.

The most prominent findings in this study are the global and regional imaging associations with DNAm CRP. To our knowledge, there are no previous studies on the relationship between peripheral blood DNA methylation CRP scores and brain structure in the context of depression. We found that DNAm CRP scores had a greater number of associations with imaging traits, and with larger effect sizes compared to serum CRP. DNAm CRP scores were associated with several structural neuroimaging measures including reductions in global grey matter and global cortical volume. We also found that increased DNAm CRP scores were associated with widespread white matter changes including reductions in FA in the external capsule and ALIC and increases in MD for projection fibres and thalamic radiations. This is highly consistent with regions implicated in previous imaging studies of MDD (van Velzen et al., 2019). The ALIC in particular, has been consistently implicated in MDD with a large body of evidence finding reduced white matter integrity in individuals with MDD (Jia et al., 2010, van Velzen et al., 2020, Zhang et al., 2013, Zhu et al., 2011). These results indicate that there may be a chronic inflammatory component to both global decreases in white matter integrity and to decreases regionally in the ALIC, as seen in MDD. Previous mendelian randomization studies have also shown that CRP is likely to be causally involved in the pathogenesis of MDD, and the alterations in brain structure we report here may represent a biologically plausible mechanism underlying the link between CRP and somatic/depressive symptoms (Khandaker et al., 2020). With increased availability of genetic instruments for brain imaging measures, future research could directly test causal and mechanistic links between inflammation, structural brain alterations and MDD.

These findings could also be considered in the context of the ‘inflammaging’ theory of ageing and accelerated ageing models of MDD (Wolkowitz et al., 2010). Chronic low-grade inflammation has been shown to accelerate age-related neurodegenerative processes including reductions in cortical volume and white matter integrity (Franceschi and Campisi, 2014, Zhao et al., 2019). Similarly, MDD is associated with increased brain atrophy and ageing-related disease and inflammation is thought to be a common biological mechanism between MDD and brain ageing (Franceschi and Campisi, 2014). We found that the DNAm score was associated with reductions in both cortical volume and white matter integrity, consistent with previous findings for both inflammation and MDD effects on brain structure, discussed above (Wersching et al., 2010). Several potential biological mechanisms have been proposed in linking immunological changes to affective neurobiology. MDD has been associated with immune cell senescence in particular, and a study by Diniz et al found that a senescence associated secretory phenotype was associated with MDD severity (Diniz et al., 2019). Future research could examine the relationships between a broader range of inflammatory markers (e.g. IL-6, HPA-axis genes), MDD and measures of biological brain age to determine the involvement of inflammatory mechanisms in accelerated brain ageing, and specifically in individuals with MDD.

Our findings indicate utility in combining serological and methylomic markers of inflammation in the investigation of MDD. Methylation markers of other traits have proven useful in the prediction of MDD in previous studies, (Humphreys et al., 2019, Ryan et al., 2017) and future work could investigate these in the context of brain imaging measures to further understanding of the role of peripheral inflammation in MDD. Our study found differential associations with MDD depending on the inflammatory marker investigated. The acute serological measure of CRP was associated with depressive symptoms whereas the chronic methylation signature demonstrated widespread associations with reduced white matter integrity of the brain. This could be due to the differential acute/chronic nature of each of these inflammatory markers. Serum CRP is a marker of current active peripheral inflammation and this was associated with current depression symptoms, particularly somatic symptoms. However, DNAm based measures, which are considered to reflect more chronic exposure over time (Byun et al., 2012), were related to what would be expected to be slower changing neural features, but not currently active, potentially transient, symptoms. Longitudinal measures of depressive symptoms may therefore be a more powerful way to detect associations with these types of chronic methylation-based inflammatory measures. This should also be considered in the context of our relatively well, community-based sample, as discussed in limitations below. From this relatively well sample, we have ascertained that chronic inflammation is robustly associated with brain atrophy and white matter disturbances, however a longitudinal study with a larger sample size of moderate/severe cases would be able to discern if chronic inflammation leading to brain atrophy is associated with longitudinal measures of MDD to disentangle these relationships further. Overall, these findings suggest a possible relationship between methylation markers of the immune system and neuroimaging traits and highlights the utility of methylomic profiles for investigating brain phenotypes.

This study has a number of strengths, in particular, the STRADL study is a large community-based sample with in-depth phenotypic assessment and neuroimaging data (n = 880 in the current study). Our study also benefits from the inclusion of methylation data which provided a long-term signature of chronic/low-level inflammation, overcoming issues with single timepoint CRP measurement which fluctuates in response to numerous factors and is prone to measurement error. We have also utilized a data-driven approach by not selecting neuroimaging regions of interest prior to analyses. This allowed an assessment of the effects of inflammation and depression on a wide range of structural neuroimaging measures.

Limitations of this study included a small number of current MDD cases in comparison to the number of controls. As this study is a community-based sample, this indicates that the participants with MDD are relatively well in comparison to MDD cases who are hospitalised. The mean depression symptom score (total QIDS) in the lifetime MDD group was 7.1, indicating mild depression levels in this group. Therefore, we are potentially not capturing the effects of moderate to severe forms of MDD. However, community-based sampling results may be more generalizable to the population than those obtained in a clinic. Furthermore, although CRP is widely used clinically to determine peripheral inflammation, it is ultimately a proxy for inflammatory activity (Stevenson et al., 2020) and the detection threshold for CRP in this study was 4 mg/L; values lower than this were recorded as 0. This could lead to missing biologically relevant interactions at lower levels of CRP. We also note that we did not separate out unipolar versus bipolar depression or investigate associations with anxiety symptoms because of limitations with sample size. Another limitation of the current study is that methylation data was collected at the GS baseline appointment. We note however that the results of the supplementary analysis accounting for this time interval between methylation data collection and imaging assessments revealed a similar pattern of findings (see Supplementary Tables 24–28). Lastly, although our study has found associations between peripheral inflammatory markers and structural brain alterations, the study does not provide evidence for causal mechanistic pathways.

In conclusion, we found that serum CRP was associated with depressive symptoms, in particular somatic symptoms, and with a reduction of entorhinal cortex thickness. This study also utilized a methylomic signature of C-reactive protein to capture chronic signatures of low-level inflammation. Here we found significant widespread associations with several structural neuroimaging measures, in particular, differences in white matter integrity, including regions previously implicated in MDD such as the external capsule and ALIC. This study highlights the utility of using both serological and methylation markers in a multi-level approach to study brain imaging and psychiatric phenotypes. Furthermore, as peripheral inflammation was associated with both changes in brain morphology and depression symptoms, inflammation may represent an important and clinically relevant therapeutic target for depressive symptoms.

## 5 Data availability

Access to and use of GS and STRADL data must be approved by the GS Access Committee under the terms of consent. Full details of the application process can be found at www.generationscotland.org.

## Funding

Generation Scotland received core support from the Chief Scientist Office of the Scottish Government Health Directorates [CZD/16/6] and the Scottish Funding Council [HR03006] and is currently supported by the Wellcome Trust [216767/Z/19/Z]. Genotyping of the GS:SFHS samples was carried out by the Genetics Core Laboratory at the Edinburgh Clinical Research Facility, University of Edinburgh, Scotland and was funded by the Medical Research Council UK and the Wellcome Trust (Wellcome Trust Strategic Award “STratifying Resilience and Depression Longitudinally” (STRADL) Reference 104036/Z/14/Z). CG is supported by The Medical Research Council and The University of Edinburgh through the Precision Medicine Doctoral Training program. SRC is supported by the UK Medical Research Council [MR/R024065/1] and a National Institutes of Health (NIH) research grant R01AG054628.

## Declaration of Competing Interest

The authors declare that they have no known competing financial interests or personal relationships that could have appeared to influence the work reported in this paper.
