# Supplementary material for: Structural brain correlates of serum and epigenetic markers of inflammation in major depressive disorder
Source: Brain Behav Immun. 2021 Feb;92:39–48. doi: 10.1016/j.bbi.2020.11.024 (PMC7910280; doi:10.1016/j.bbi.2020.11.024)
Supplement: Supplementary data 1 [file mmc1.docx]

**Supplemental Materials**

**STRADL MRI Parameters**

The structural sequences collected were as follows: 3D T1-weighted fast gradient echo with magnetisation preparation; 3D T2-weighted fast spin echo; 3D Fluid Attenuation Inversion Recovery (FLAIR); Diffusion Tensor Imaging (DTI); and Susceptibility Weighted Imaging (SWI) or T2*-weighted gradient echo (Habota et al., 2019). Participants in Dundee were scanned using a Siemens 3T Prisma-FIT (Siemens Healthineers, Erlangen, Germany) with a 20-channel head and neck coil and a back-facing mirror (software version VE11, gradient with max amplitude 80 mT/m and maximum slew rate 200 T/m/s). In Aberdeen, participants were imaged on a 3T Philips Achieva TX series MRI system (Philips Healthcare, Best, Netherlands) with a 32-channel phased-array head coil with a back-facing mirror (software version 5.1.7; gradients with maximum amplitude 80 mT/m and maximum slew rate 100 T/m/s; Habota et al., 2019).

**Freesurfer Quality Control**

All scans were visually assessed for parcellation errors and participants with any major errors in segmentation or cortical parcellation were excluded from analyses. Manual brain mask edits were made when the skull was included in parcellations to remove the skull from the parcellated tissue. Further edits were made if parts of the brain were not included in the parcellation such that we manually delineated the boundaries to include the missing tissue. A record of scan edits was kept and participants were given a score of 0 if they had no manual edits to their scan or a score of 1 if they had either an edit to remove skull, a white matter edit or a parcellation edit. This was recorded as a covariate to use in statistical analyses since the process of QC and manual editing of scans is a subjective process which could introduce bias, this covariate would account for potential differences between unedited and edited scans.

**DTI Quality Control**

QC was performed following ENIGMA DTI protocols (<http://enigma.ini.usc.edu/protocols/dti-protocols/>). Briefly this included (1) correcting for eddy current-induced distortions and subject movement in the scanner; (2) skull stripping using BET at a threshold of 0.2; (3) using DTIFIT in order to compute diffusion tensor characteristics (i.e. principal eigenvectors or V1, V2, V3; eigenvalues or L1, L2, L3; fractional anisotropy (FA), mean diffusivity (MD); and (4) visually checking the quality of FA images at this stage in order to exclude distorted images. There were also three tracts not included in any tract categories: corpus callosum, corona radiata and internal capsule. The 5 unilateral tracts included the corpus callosum, fornix and the body, genu and splenium of the corpus callosum.

**Table S1:** Variables included in structural brain analyses and how they were derived

| **Variable** | **Measures** |
| --- | --- |
| **Global Measures:** |  |
| Global Cortical Volume | Sum of 5 lobar measures |
| Global Cortical Thickness | Weighted average of thickness of the 5 lobes multiplied by their surface area and divided by their thickness |
| Global Surface Area | Sum of 5 lobar measures |
| **Lobes:** |  |
| Frontal | Sum of: Superior frontal gyrus, Rostral middle frontal, caudal middle frontal, Pars orbitalis, pars triangularis, pars opercularis, Frontal pole, Lateral orbitofrontal, medial orbitofrontal, Precentral gyrus, paracentral cortex |
| Temporal | Sum of: Insula, Superior temporal, transverse temporal, banks STS, Middle temporal gyrus, Inferior temporal gyrus, Temporal pole, Fusiform, parahippocampal, entorhinal |
| Parietal | Sum of: Postcentral gyrus, paracentral cortex, Superior parietal cortex, Inferior parietal cortex, Supramarginal gyrus, Precuneus |
| Occipital | Sum of: Lateral occipital cortex, Cuneus, Pericalcarine cortex, Lingual gyrus |
| Cingulate | Sum of: Rostral ACC, Caudal ACC, Posterior cingulate cortex, Cingulate isthmus |
| **Individual Structures:** |  |
| 34 Cortical Regions | All of the above in the lobe categories |
| 7 Subcortical | Nucelus accumbens, amygdala, caudate nucleus, hippocampus, pallidum, putamen and thalamus |
| **General DTI Measures** |  |
| gFA/MD | PCA of all tracts excluding the Corpus Callosum, Corona Radiata and Internal Capsule as their subsections were included. |
| Average FA/MD | Average of all tracts |
| gAssociation Fibres FA/MD | PCA of 10 tracts; Cingulum-Cingulate Gyrus, Cingulum-Hippocampus, External Capsule, Fornix- Column&Body, Fornix-Cres/Stria Terminalis, Inferior fronto-occipital fasciculus, Superior fronto-occipital fasciculus, Superior longitudinal fasciculus, Sagittal Striatum (includes inf. longitudinal fasciculus and inferior-fronto-occipital fasciculus) and Uncinate Fasciculus |
| gCommisural Fibres FA/MD | PCA of 3 tracts; Body of corpus callosum, Genu of corpus callosum and splenium of corpus callosum |
| gProjection Fibres FA/MD | PCA of 8 tracts; Anterior corona radiata, Corticospinal tract, Posterior corona radiata, Posterior limb of internal capsule, Superior corona radiata, Anterior limb of internal capsule. Posterior thalamic radiation and Rentrolenticular part of internal capsule |
| gThalamic Radiations FA/MD | PCA of 4 tracts; Anterior Limb of Internal Capsule, Posterior Thalamic Radiation, Retrolenticular part of internal capsule and Posterior limb of internal capsule |
| Individual Tracts | - |

**Table S2**: Variance explained by the first principal component for DTI PCA

| **PCA** | **FA** | **MD** |
| --- | --- | --- |
| gTotal | 45.2% | 61.9% |
| Association Fibres | 45.5% | 72.9% |
| Commisural Fibres | 79.6% | 84.7% |
| Projection Fibres | 54% | 67.1% |
| Thalamic Radiations | 64.7% | 72.1% |

| **Measure** | **(1) Serum CRP** | | | **(2) DNAm CRP- Minimally Adjusted** | | | **(3) DNAm CRP- Fully Adjusted** | | |
| --- | --- | --- | --- | --- | --- | --- | --- | --- | --- |
|  | **β** | **SE** | **pFDR** | **β** | **SE** | **pFDR** | **β** | **SE** | **pFDR** |
| **Total Score** |  |  |  |  |  |  |  |  |  |
| MDD Case Control Status | 0.010 | 0.034 | 0.764 | 0.072 | 0.043 | 0.093 | 0.025 | 0.045 | 0.574 |
| Total QIDS Score | 0.073 | 0.034 | **0.033** | 0.106 | 0.044 | **0.016** | 0.005 | 0.044 | 0.914 |
| **Individual QIDS Item** |  |  |  |  |  |  |  |  |  |
| QIDS1- Falling asleep | 0.092 | 0.035 | 0.051 | 0.009 | 0.044 | 0.908 | -0.032 | 0.046 | 0.892 |
| QIDS2- Sleeping during the night | -0.040 | 0.035 | 0.471 | 0.025 | 0.044 | 0.763 | 0.006 | 0.047 | 0.892 |
| QIDS3- Waking up too early | 0.015 | 0.036 | 0.725 | -0.031 | 0.045 | 0.704 | -0.049 | 0.047 | 0.892 |
| QIDS4- Sleeping too much | 0.062 | 0.036 | 0.236 | -0.003 | 0.045 | 0.955 | -0.013 | 0.047 | 0.892 |
| QIDS5- Feeling sad | 0.043 | 0.035 | 0.432 | 0.103 | 0.044 | 0.126 | 0.016 | 0.046 | 0.892 |
| QIDS6- Decreased appetite | 0.027 | 0.044 | 0.725 | 0.093 | 0.054 | 0.232 | 0.047 | 0.057 | 0.892 |
| QIDS7- Increased appetite | -0.018 | 0.049 | 0.725 | 0.012 | 0.066 | 0.908 | -0.049 | 0.062 | 0.892 |
| QIDS8- Decreased weight | 0.024 | 0.048 | 0.725 | 0.089 | 0.055 | 0.251 | 0.036 | 0.056 | 0.892 |
| QIDS9- Increased weight | -0.017 | 0.047 | 0.725 | 0.132 | 0.066 | 0.149 | 0.077 | 0.067 | 0.892 |
| QIDS10- Concentration/decision making | 0.013 | 0.036 | 0.725 | 0.021 | 0.044 | 0.785 | -0.027 | 0.047 | 0.892 |
| QIDS11- View of myself | 0.047 | 0.036 | 0.418 | 0.046 | 0.044 | 0.480 | -0.006 | 0.046 | 0.892 |
| QIDS12- Thoughts of suicide or death | 0.064 | 0.036 | 0.236 | 0.056 | 0.045 | 0.425 | -0.008 | 0.046 | 0.892 |
| QIDS13- General interest | 0.145 | 0.035 | **0.001** | 0.096 | 0.044 | 0.126 | 0.016 | 0.046 | 0.892 |
| QIDS14- Energy level | 0.101 | 0.034 | **0.027** | 0.095 | 0.044 | 0.126 | 0.021 | 0.045 | 0.892 |
| QIDS15- Feeling slowed down | 0.085 | 0.036 | 0.068 | 0.108 | 0.044 | 0.126 | 0.079 | 0.046 | 0.892 |
| QIDS16- Feeling restless | 0.035 | 0.036 | 0.532 | 0.051 | 0.044 | 0.439 | -0.009 | 0.047 | 0.892 |

**Table S3:** Results of MDD and QIDS symptoms analyses for (1) Serum CRP, (2) DNAm CRP minimally adjusted and (3) DNAm CRP fully-adjusted. P_FDR_ significant associations are marked in bold.

Covariates: (1) age, sex, assessment centre, BMI; (2) age, sex, assessment centre, methylation set; (3) age, sex, assessment centre, methylation set, smoking status, pack years, BMI.

**Tabulated Results:**

**Table S4-S9**: Serum CRP and DNAm CRP associations with structural brain measures

**Table S10-S15**: Serum CRP/ DNAm CRP interaction effects with MDD case control status on structural brain measures

In the above tables all serum CRP analyses are controlled for: age, sex, assessment centre and BMI and hemisphere for bilateral structures. T1 data is additionally controlled for imaging batch, intracranial volume and image edits. P_FDR_ significant associations are marked in bold.

All DNAm results reported are fully adjusted models controlling for age, sex, assessment centre, BMI, smoking status, pack years, methylation set and hemisphere for bilateral structures. Again, T1 data included imaging batch, intracranial volume and number of image edits as additional covariates. P_FDR_ significant associations are marked in bold.

**Supplementary Analyses:**

**Table S16- S21**: MDD/ Total QIDS associations with structural brain measures

MDD/QIDS analyses are controlled for age, sex, assessment centre and hemisphere for bilateral structures. T1 data included imaging batch, intracranial volume and number of image edits as additional covariates. P_FDR_ significant associations are marked in bold.

**Table S22**- Serum CRP associations with MDD additionally controlling for concurrent smoking, age, sex, BMI, site. P_FDR_ significant associations are marked in bold.

**Table S23**- Serum CRP associations with clinical features of MDD (age of onset (<= or > 21 years, recurrence, QIDS severity). Covariates- age, sex, BMI, site. P_FDR_ significant associations are marked in bold.

**Table S24-S28**: DNAm CRP associations with structural brain measures additionally controlling for methylation time difference. Covariates- age, sex, site, methylation set, BMI, smoking status, pack years, time difference (days) plus relevant imaging covariates described above. P_FDR_ significant associations are marked in bold.

|  | **Structural Phenotype** | **Serum CRP** | | | **DNAm CRP** | | |
| --- | --- | --- | --- | --- | --- | --- | --- |
|  |  | **β** | **SE** | **pFDR** | **β** | **SE** | **pFDR** |
| **Global Brain Measures** | Global Total Grey Matter | -0.004 | 0.019 | 0.838 | -0.056 | 0.024 | **0.018** |
|  | Global Cerebral White Matter | 0.001 | 0.024 | 0.957 | -0.057 | 0.031 | 0.068 |
|  | Global Cortical Volume | -0.041 | 0.031 | 0.177 | -0.099 | 0.039 | **0.012** |
|  | Global Cortical Thickness | -0.002 | 0.032 | 0.954 | -0.052 | 0.039 | 0.180 |
|  | Global Cortical Surface Area | -0.018 | 0.032 | 0.567 | -0.071 | 0.042 | 0.095 |
|  | Intracranial Volume | -0.029 | 0.030 | 0.334 | 0.009 | 0.039 | 0.820 |
| **Lobar Volume** | Frontal Lobe Volume | -0.001 | 0.022 | 0.978 | -0.067 | 0.028 | 0.086 |
|  | Temporal Lobe Volume | -0.021 | 0.023 | 0.978 | -0.052 | 0.029 | 0.098 |
|  | Parietal Lobe Volume | -0.002 | 0.024 | 0.978 | -0.054 | 0.030 | 0.098 |
|  | Occipital Lobe Volume | -0.014 | 0.027 | 0.978 | -0.067 | 0.034 | 0.098 |
|  | Cingulate Lobe Volume | -0.021 | 0.029 | 0.978 | -0.042 | 0.036 | 0.247 |
| **Lobar Thickness** | Frontal Lobe Thickness | -0.016 | 0.033 | 0.714 | -0.055 | 0.041 | 0.330 |
|  | Temporal Lobe Thickness | -0.039 | 0.033 | 0.714 | -0.050 | 0.040 | 0.330 |
|  | Parietal Lobe Thickness | 0.020 | 0.029 | 0.714 | -0.026 | 0.036 | 0.479 |
|  | Occipital Lobe Thickness | 0.012 | 0.033 | 0.714 | -0.045 | 0.040 | 0.330 |
|  | Cingulate Lobe Thickness | -0.033 | 0.036 | 0.714 | -0.058 | 0.045 | 0.330 |
| **Lobar Surface Area** | Frontal Lobe Surface Area | 0.013 | 0.025 | 0.923 | -0.052 | 0.031 | 0.184 |
|  | Temporal Lobe Surface Area | -0.002 | 0.025 | 0.923 | -0.045 | 0.031 | 0.184 |
|  | Parietal Lobe Surface Area | -0.007 | 0.026 | 0.923 | -0.050 | 0.032 | 0.184 |
|  | Occipital Lobe Surface Area | -0.019 | 0.028 | **0.923** | -0.057 | 0.036 | 0.184 |
|  | Cingulate Lobe Surface Area | -0.008 | 0.028 | 0.923 | 0.008 | 0.035 | 0.830 |

**Table S4: Global and lobar associations with serum CRP and DNAm CRP**

| **Individual Brain Structure** | **Cortical Volume** | | | **Cortical Surface Area** | | | **Cortical Thickness** | | |
| --- | --- | --- | --- | --- | --- | --- | --- | --- | --- |
|  | **Serum CRP** | | | **Serum CRP** | | | **Serum CRP** | | |
|  | **β** | **SE** | **pFDR** | **β** | **SE** | **pFDR** | **β** | **SE** | **pFDR** |
| **Bank Superior Temporal Sulcus** | -0.011 | 0.029 | 0.993 | -0.005 | 0.029 | 0.982 | -0.004 | 0.031 | 0.963 |
| **Caudal Anterior Cingulate** | -0.025 | 0.026 | 0.970 | -0.022 | 0.027 | 0.982 | 0.004 | 0.031 | 0.963 |
| **Caudal Middle Frontal** | 0.039 | 0.030 | 0.966 | 0.032 | 0.030 | 0.982 | 0.023 | 0.031 | 0.865 |
| **Cuneus** | -0.023 | 0.030 | 0.993 | -0.022 | 0.030 | 0.982 | -0.025 | 0.031 | 0.865 |
| **Entorhinal Cortex** | -0.075 | 0.028 | 0.243 | -0.020 | 0.029 | 0.982 | -0.105 | 0.032 | **0.043** |
| **Frontal Pole** | -0.069 | 0.029 | 0.316 | -0.073 | 0.028 | 0.335 | -0.024 | 0.031 | 0.865 |
| **Fusiform** | -0.003 | 0.027 | 0.993 | -0.009 | 0.027 | 0.982 | -0.020 | 0.033 | 0.865 |
| **Inferior Parietal** | -0.054 | 0.027 | 0.551 | -0.048 | 0.028 | 0.982 | 0.002 | 0.030 | 0.980 |
| **Inferior Temporal** | -0.029 | 0.026 | 0.966 | -0.034 | 0.027 | 0.982 | -0.004 | 0.030 | 0.963 |
| **Insula** | 0.003 | 0.026 | 0.993 | 0.025 | 0.026 | 0.982 | -0.046 | 0.031 | 0.865 |
| **Isthmus Cingulate** | 0.016 | 0.029 | 0.993 | 0.033 | 0.027 | 0.982 | -0.019 | 0.031 | 0.865 |
| **Lateral Occipital** | 0.008 | 0.027 | 0.993 | -0.010 | 0.028 | 0.982 | 0.026 | 0.031 | 0.865 |
| **Lateral Orbito Frontal** | -0.017 | 0.026 | 0.993 | -0.003 | 0.028 | 0.982 | -0.031 | 0.034 | 0.865 |
| **Lingual** | -0.037 | 0.030 | 0.966 | -0.019 | 0.031 | 0.982 | -0.014 | 0.033 | 0.865 |
| **Medial Orbito Frontal** | -0.030 | 0.025 | 0.966 | -0.023 | 0.026 | 0.982 | 0.000 | 0.032 | 0.993 |
| **Middle Temporal** | -0.025 | 0.025 | 0.970 | -0.006 | 0.027 | 0.982 | -0.030 | 0.030 | 0.865 |
| **Paracentral** | -0.030 | 0.028 | 0.966 | -0.015 | 0.028 | 0.982 | -0.033 | 0.030 | 0.865 |
| **Parahippocampal** | -0.020 | 0.031 | 0.993 | -0.011 | 0.029 | 0.982 | -0.015 | 0.034 | 0.865 |
| **Pars Opercularis** | -0.009 | 0.029 | 0.993 | 0.005 | 0.029 | 0.982 | -0.040 | 0.029 | 0.865 |
| **Pars Orbitalis** | -0.046 | 0.027 | 0.721 | -0.036 | 0.028 | 0.982 | -0.023 | 0.030 | 0.865 |
| **Pars Traingularis** | -0.002 | 0.029 | 0.993 | -0.002 | 0.030 | 0.982 | -0.009 | 0.031 | 0.960 |
| **Pericalcarine** | -0.020 | 0.032 | 0.993 | -0.016 | 0.033 | 0.982 | -0.016 | 0.033 | 0.865 |
| **Post Central** | -0.014 | 0.028 | 0.993 | -0.014 | 0.028 | 0.982 | 0.006 | 0.029 | 0.963 |
| **Posterior Cingulate** | -0.016 | 0.028 | 0.993 | 0.002 | 0.027 | 0.982 | -0.039 | 0.032 | 0.865 |
| **Precentral** | -0.002 | 0.029 | 0.993 | 0.002 | 0.028 | 0.982 | -0.014 | 0.033 | 0.865 |
| **Precuneus** | -0.003 | 0.027 | 0.993 | 0.014 | 0.028 | 0.982 | -0.014 | 0.031 | 0.865 |
| **Rostral Anterior Cingulate** | -0.040 | 0.027 | 0.966 | -0.027 | 0.027 | 0.982 | -0.034 | 0.031 | 0.865 |
| **Rostral Middle Frontal** | -0.010 | 0.026 | 0.993 | -0.001 | 0.028 | 0.982 | -0.015 | 0.031 | 0.865 |
| **Superior Frontal** | 0.017 | 0.025 | 0.993 | 0.017 | 0.026 | 0.982 | -0.009 | 0.033 | 0.960 |
| **Superior Parietal** | 0.003 | 0.028 | 0.993 | -0.018 | 0.029 | 0.982 | 0.023 | 0.029 | 0.865 |
| **Superior Temporal** | 0.000 | 0.027 | 0.993 | 0.023 | 0.027 | 0.982 | -0.031 | 0.032 | 0.865 |
| **Supramarginal** | 0.008 | 0.027 | 0.993 | 0.005 | 0.027 | 0.982 | 0.013 | 0.030 | 0.865 |
| **Temporal Pole** | -0.012 | 0.030 | 0.993 | -0.007 | 0.028 | 0.982 | -0.031 | 0.033 | 0.865 |
| **Transverse Temporal** | 0.001 | 0.031 | 0.993 | 0.021 | 0.031 | 0.982 | -0.053 | 0.032 | 0.865 |

**Table S5: Serum CRP associations with individual cortical brain structures (volume, surface area and thickness)**

| **Individual Brain Structure** | **Cortical Volume** | | | **Cortical Surface Area** | | | **Cortical Thickness** | | |
| --- | --- | --- | --- | --- | --- | --- | --- | --- | --- |
|  | **DNAm CRP** | | | **DNAm CRP** | | | **DNAm CRP** | | |
|  | **β** | **SE** | **pFDR** | **β** | **SE** | **pFDR** | **β** | **SE** | **pFDR** |
| **Bank Superior Temporal Sulcus** | -0.051 | 0.037 | 0.506 | -0.054 | 0.037 | 0.431 | -0.065 | 0.039 | 0.267 |
| **Caudal Anterior Cingulate** | -0.027 | 0.033 | 0.635 | -0.008 | 0.034 | 0.891 | -0.015 | 0.038 | 0.832 |
| **Caudal Middle Frontal** | -0.062 | 0.036 | 0.356 | -0.065 | 0.037 | 0.431 | -0.044 | 0.039 | 0.490 |
| **Cuneus** | -0.041 | 0.038 | 0.592 | -0.077 | 0.038 | 0.431 | -0.003 | 0.040 | 0.945 |
| **Entorhinal Cortex** | -0.028 | 0.036 | 0.635 | -0.057 | 0.037 | 0.431 | 0.028 | 0.041 | 0.718 |
| **Frontal Pole** | 0.006 | 0.037 | 0.969 | -0.052 | 0.036 | 0.431 | 0.087 | 0.038 | 0.143 |
| **Fusiform** | -0.086 | 0.035 | 0.112 | -0.057 | 0.035 | 0.431 | -0.085 | 0.041 | 0.161 |
| **Inferior Parietal** | -0.031 | 0.034 | 0.597 | -0.017 | 0.035 | 0.797 | -0.074 | 0.037 | 0.161 |
| **Inferior Temporal** | -0.063 | 0.033 | 0.290 | -0.086 | 0.034 | 0.227 | -0.005 | 0.038 | 0.945 |
| **Insula** | -0.008 | 0.031 | 0.932 | -0.020 | 0.032 | 0.783 | -0.056 | 0.039 | 0.360 |
| **Isthmus Cingulate** | -0.027 | 0.036 | 0.652 | -0.002 | 0.034 | 0.973 | -0.046 | 0.039 | 0.490 |
| **Lateral Occipital** | -0.070 | 0.034 | 0.233 | -0.043 | 0.035 | 0.480 | -0.070 | 0.039 | 0.221 |
| **Lateral Orbito Frontal** | -0.002 | 0.033 | 0.984 | -0.002 | 0.036 | 0.973 | -0.015 | 0.042 | 0.832 |
| **Lingual** | -0.062 | 0.038 | 0.356 | -0.051 | 0.039 | 0.439 | -0.041 | 0.041 | 0.510 |
| **Medial Orbito Frontal** | 0.003 | 0.032 | 0.979 | -0.023 | 0.033 | 0.743 | 0.044 | 0.040 | 0.490 |
| **Middle Temporal** | -0.017 | 0.031 | 0.808 | -0.050 | 0.033 | 0.431 | -0.003 | 0.037 | 0.945 |
| **Paracentral** | -0.036 | 0.036 | 0.597 | -0.034 | 0.035 | 0.607 | -0.055 | 0.037 | 0.360 |
| **Parahippocampal** | -0.048 | 0.039 | 0.543 | 0.015 | 0.037 | 0.801 | -0.103 | 0.043 | 0.138 |
| **Pars Opercularis** | 0.016 | 0.036 | 0.866 | 0.048 | 0.037 | 0.439 | -0.105 | 0.037 | 0.052 |
| **Pars Orbitalis** | -0.081 | 0.033 | 0.112 | -0.059 | 0.034 | 0.431 | -0.013 | 0.038 | 0.832 |
| **Pars Traingularis** | 0.000 | 0.036 | 0.997 | 0.016 | 0.038 | 0.801 | -0.014 | 0.039 | 0.832 |
| **Pericalcarine** | -0.013 | 0.041 | 0.921 | -0.033 | 0.041 | 0.694 | -0.015 | 0.042 | 0.832 |
| **Post Central** | -0.034 | 0.036 | 0.597 | -0.049 | 0.035 | 0.431 | -0.015 | 0.037 | 0.832 |
| **Posterior Cingulate** | -0.041 | 0.035 | 0.543 | -0.009 | 0.034 | 0.891 | -0.037 | 0.039 | 0.530 |
| **Precentral** | -0.112 | 0.036 | 0.066 | -0.051 | 0.035 | 0.431 | -0.117 | 0.040 | 0.052 |
| **Precuneus** | -0.056 | 0.034 | 0.356 | -0.059 | 0.034 | 0.431 | -0.041 | 0.038 | 0.491 |
| **Rostral Anterior Cingulate** | -0.032 | 0.034 | 0.597 | 0.001 | 0.035 | 0.973 | -0.043 | 0.039 | 0.490 |
| **Rostral Middle Frontal** | -0.087 | 0.032 | 0.112 | -0.093 | 0.035 | 0.227 | -0.014 | 0.039 | 0.832 |
| **Superior Frontal** | -0.072 | 0.031 | 0.147 | -0.036 | 0.033 | 0.553 | -0.090 | 0.040 | 0.143 |
| **Superior Parietal** | -0.041 | 0.034 | 0.543 | -0.038 | 0.036 | 0.564 | -0.049 | 0.037 | 0.413 |
| **Superior Temporal** | -0.032 | 0.034 | 0.597 | 0.016 | 0.034 | 0.797 | -0.076 | 0.039 | 0.178 |
| **Supramarginal** | -0.039 | 0.033 | 0.543 | -0.018 | 0.034 | 0.797 | -0.076 | 0.037 | 0.161 |
| **Temporal Pole** | 0.006 | 0.038 | 0.969 | -0.021 | 0.035 | 0.783 | -0.006 | 0.040 | 0.945 |
| **Transverse Temporal** | -0.015 | 0.039 | 0.873 | 0.034 | 0.039 | 0.641 | -0.118 | 0.041 | 0.052 |

**Table S6: DNAm CRP associations with individual cortical brain structures (volume, surface area and thickness)**

| **Subcortical Volume** | **(1) Serum CRP** | | | **(2) DNAm CRP** | | |
| --- | --- | --- | --- | --- | --- | --- |
|  | **β** | **SE** | **pFDR** | **β** | **SE** | **pFDR** |
| **Nucleus Accumbens** | 0.037 | 0.029 | 0.353 | 0.003 | 0.037 | 0.965 |
| **Amygdala** | 0.049 | 0.029 | 0.353 | -0.008 | 0.037 | 0.965 |
| **Caudate** | 0.039 | 0.031 | 0.353 | -0.002 | 0.039 | 0.965 |
| **Hippocampus** | 0.038 | 0.030 | 0.353 | 0.013 | 0.038 | 0.965 |
| **Pallidum** | 0.048 | 0.029 | 0.353 | -0.026 | 0.037 | 0.965 |
| **Putamen** | 0.015 | 0.030 | 0.622 | -0.011 | 0.038 | 0.965 |
| **Thalamus** | 0.012 | 0.024 | 0.622 | -0.013 | 0.030 | 0.965 |
| **Ventral Diencephalon** | 0.020 | 0.027 | 0.605 | -0.023 | 0.034 | 0.965 |

**Table S7: Serum CRP and DNAm CRP associations with subcortical volumes**

|  | **DTI Measure** | **Serum CRP** | | | **DNAm CRP** | | |
| --- | --- | --- | --- | --- | --- | --- | --- |
|  |  | **β** | **SE** | **pFDR** | **β** | **SE** | **pFDR** |
| **Mean Diffusivity** | gMD | -0.018 | 0.033 | 0.580 | 0.072 | 0.041 | 0.081 |
|  | Average MD | -0.003 | 0.030 | 0.922 | 0.122 | 0.037 | **0.001** |
|  | Association Fibres | -0.021 | 0.033 | 0.696 | 0.063 | 0.042 | 0.132 |
|  | Commisural Fibres | 0.006 | 0.031 | 0.857 | 0.080 | 0.039 | 0.053 |
|  | Projection Fibres | 0.019 | 0.029 | 0.696 | 0.098 | 0.035 | **0.019** |
|  | Thalamic Radiations | 0.020 | 0.027 | 0.696 | 0.079 | 0.032 | **0.028** |
| **Fractional Anisotropy** | gFA | 0.027 | 0.030 | 0.367 | -0.076 | 0.038 | **0.046** |
|  | Average FA | 0.004 | 0.031 | 0.901 | -0.092 | 0.039 | **0.020** |
|  | Association Fibres | 0.035 | 0.031 | 0.633 | -0.091 | 0.038 | 0.069 |
|  | Commisural Fibres | 0.015 | 0.032 | 0.633 | -0.043 | 0.040 | 0.312 |
|  | Projection Fibres | 0.020 | 0.031 | 0.633 | -0.044 | 0.040 | 0.312 |
|  | Thalamic Radiations | 0.027 | 0.030 | 0.633 | -0.039 | 0.038 | 0.312 |

**Table S8: Serum CRP and DNAm CRP associations with global DTI measures**

|  | **Serum CRP** | | | | | | **DNAm CRP** | | | | | |
| --- | --- | --- | --- | --- | --- | --- | --- | --- | --- | --- | --- | --- |
| **White Matter Tract** | **Mean Diffusivity** | | | **Fractional Anisotropy** | | | **Mean Diffusivity** | | | **Fractional Anisotropy** | | |
|  | **β** | **SE** | **pFDR** | **β** | **SE** | **pFDR** | **β** | **SE** | **pFDR** | **β** | **SE** | **pFDR** |
| Anterior Corona Radiata | -0.0028 | 0.0349 | 0.9860 | 0.0223 | 0.0318 | 0.9267 | 0.0681 | 0.0423 | 0.1336 | -0.059 | 0.040 | 0.394 |
| Anterior Limb Internal Capsule | 0.0387 | 0.0331 | 0.9860 | 0.0123 | 0.0337 | 0.9267 | 0.1000 | 0.0391 | **0.0420** | -0.121 | 0.042 | **0.048** |
| Cingulum (cingulate gyrus) | -0.0027 | 0.0292 | 0.9860 | 0.0100 | 0.0324 | 0.9267 | 0.0814 | 0.0367 | **0.0436** | -0.052 | 0.041 | 0.394 |
| Cingulum (hippocampus) | 0.0009 | 0.0244 | 0.9860 | 0.0103 | 0.0280 | 0.9267 | 0.0171 | 0.0297 | 0.5704 | -0.035 | 0.035 | 0.462 |
| Corona Radiata | 0.0041 | 0.0338 | 0.9860 | -0.0074 | 0.0328 | 0.9267 | 0.0911 | 0.0395 | **0.0420** | -0.050 | 0.041 | 0.394 |
| Corticospinal Tract | 0.0413 | 0.0312 | 0.9860 | 0.0347 | 0.0318 | 0.9267 | 0.0912 | 0.0389 | **0.0420** | -0.014 | 0.040 | 0.759 |
| External Capsule | -0.0006 | 0.0316 | 0.9860 | 0.0580 | 0.0324 | 0.8905 | 0.1229 | 0.0393 | **0.0225** | -0.136 | 0.040 | **0.017** |
| Fornix(Cres)/ Stria Terminalis | 0.0191 | 0.0263 | 0.9860 | 0.0212 | 0.0273 | 0.9267 | 0.0789 | 0.0321 | **0.0420** | -0.032 | 0.035 | 0.490 |
| Internal Capsule | 0.0291 | 0.0267 | 0.9860 | 0.0235 | 0.0295 | 0.9267 | 0.0717 | 0.0318 | **0.0420** | -0.048 | 0.038 | 0.394 |
| Inferior- Fronto-Occipital Fasciculus | -0.0135 | 0.0297 | 0.9860 | 0.0697 | 0.0318 | 0.6900 | 0.0841 | 0.0373 | **0.0420** | -0.097 | 0.040 | 0.127 |
| Posterior Corona Radiata | 0.0177 | 0.0310 | 0.9860 | -0.0114 | 0.0331 | 0.9267 | 0.1188 | 0.0357 | **0.0225** | -0.030 | 0.041 | 0.594 |
| Posterior Limb of Internal Capsule | 0.0333 | 0.0254 | 0.9860 | 0.0188 | 0.0289 | 0.9267 | 0.0177 | 0.0311 | 0.5704 | 0.017 | 0.037 | 0.707 |
| Posterior Thalamic Radiation | -0.0052 | 0.0301 | 0.9860 | 0.0239 | 0.0305 | 0.9267 | 0.0769 | 0.0374 | 0.0565 | -0.019 | 0.038 | 0.697 |
| Retrolenticular part of Internal Capsule | -0.0032 | 0.0250 | 0.9860 | 0.0272 | 0.0282 | 0.9267 | 0.0747 | 0.0302 | **0.0420** | -0.043 | 0.036 | 0.394 |
| Superior Corona Radiata | 0.0035 | 0.0328 | 0.9860 | -0.0370 | 0.0345 | 0.9267 | 0.0832 | 0.0369 | **0.0420** | -0.025 | 0.043 | 0.682 |
| Superior Fronto-Occipital Fasciculus | 0.0146 | 0.0328 | 0.9860 | -0.0039 | 0.0317 | 0.9267 | 0.0717 | 0.0390 | 0.0889 | -0.077 | 0.040 | 0.252 |
| Superior Longitudinal Fasciculus | 0.0018 | 0.0306 | 0.9860 | 0.0031 | 0.0335 | 0.9267 | 0.0922 | 0.0374 | **0.0420** | -0.047 | 0.041 | 0.398 |
| Sagittal Striatum | -0.0099 | 0.0296 | 0.9860 | 0.0048 | 0.0304 | 0.9267 | 0.0995 | 0.0364 | **0.0420** | -0.084 | 0.038 | 0.158 |
| Uncinate Fasciculus | 0.0229 | 0.0333 | 0.9860 | 0.0043 | 0.0337 | 0.9267 | 0.0942 | 0.0392 | **0.0420** | -0.045 | 0.041 | 0.398 |
| Body Corpus Callosum | 0.0228 | 0.0334 | 0.9860 | -0.0055 | 0.0352 | 0.9267 | 0.0969 | 0.0415 | **0.0420** | -0.061 | 0.044 | 0.394 |
| Corpus Callosum | 0.0085 | 0.0313 | 0.9860 | 0.0109 | 0.0320 | 0.9267 | 0.0832 | 0.0389 | **0.0495** | -0.050 | 0.040 | 0.394 |
| Fornix (column and body) | -0.0242 | 0.0335 | 0.9860 | 0.0408 | 0.0318 | 0.9267 | 0.0520 | 0.0419 | 0.2340 | -0.061 | 0.040 | 0.394 |
| Genu Corpus Callosum | -0.0045 | 0.0323 | 0.9860 | 0.0298 | 0.0320 | 0.9267 | 0.0635 | 0.0404 | 0.1336 | -0.012 | 0.041 | 0.762 |
| Splenium of Corpus Callosum | -0.0010 | 0.0294 | 0.9860 | 0.0173 | 0.0278 | 0.9267 | 0.0580 | 0.0369 | 0.1336 | -0.042 | 0.035 | 0.394 |

**Table S9: Serum CRP and DNAm CRP associations with individual white matter tracts.**

|  | **Structural Phenotype** | **Serum CRP* MDD** | | | **DNAm CRP*MDD** | | |
| --- | --- | --- | --- | --- | --- | --- | --- |
|  |  | **β** | **SE** | **pFDR** | **β** | **SE** | **pFDR** |
| **Global Brain Measures** | Global Total Grey Matter | 0.032 | 0.037 | 0.390 | -0.013 | 0.048 | 0.787 |
|  | Global Cerebral White Matter | 0.036 | 0.048 | 0.458 | 0.047 | 0.063 | 0.458 |
|  | Global Cortical Volume | -0.072 | 0.061 | 0.236 | -0.101 | 0.080 | 0.204 |
|  | Global Cortical Thickness | 0.009 | 0.063 | 0.887 | -0.090 | 0.078 | 0.249 |
|  | Global Cortical Surface Area | 0.019 | 0.064 | 0.766 | 0.059 | 0.086 | 0.491 |
| **Lobar Volume** | Frontal Lobe Volume | -0.009 | 0.045 | 0.832 | -0.061 | 0.057 | 0.898 |
|  | Temporal Lobe Volume | 0.062 | 0.046 | 0.832 | 0.029 | 0.060 | 0.898 |
|  | Parietal Lobe Volume | 0.036 | 0.047 | 0.832 | 0.008 | 0.062 | 0.898 |
|  | Occipital Lobe Volume | 0.025 | 0.053 | 0.832 | 0.010 | 0.070 | 0.898 |
|  | Cingulate Lobe Volume | 0.016 | 0.057 | 0.832 | -0.016 | 0.074 | 0.898 |
| **Lobar Thickness** | Frontal Lobe Thickness | -0.030 | 0.065 | 0.873 | -0.101 | 0.082 | 0.754 |
|  | Temporal Lobe Thickness | -0.010 | 0.065 | 0.873 | -0.005 | 0.082 | 0.951 |
|  | Parietal Lobe Thickness | 0.020 | 0.058 | 0.873 | -0.076 | 0.074 | 0.754 |
|  | Occipital Lobe Thickness | 0.065 | 0.065 | 0.846 | -0.061 | 0.083 | 0.765 |
|  | Cingulate Lobe Thickness | -0.068 | 0.071 | 0.846 | 0.010 | 0.092 | 0.951 |
| **Lobar Surface Area** | Frontal Lobe Surface Area | 0.011 | 0.049 | 0.856 | -0.007 | 0.062 | 0.906 |
|  | Temporal Lobe Surface Area | 0.087 | 0.048 | 0.356 | 0.035 | 0.063 | 0.906 |
|  | Parietal Lobe Surface Area | 0.037 | 0.050 | 0.777 | 0.077 | 0.065 | 0.906 |
|  | Occipital Lobe Surface Area | -0.010 | 0.056 | 0.856 | 0.018 | 0.074 | 0.906 |
|  | Cingulate Lobe Surface Area | 0.046 | 0.055 | 0.777 | -0.012 | 0.071 | 0.906 |

**Table S10: Global and lobar interaction effects with serum CRP/DNAm CRP and MDD Case/Control Status**

| **Individual Brain Structure** | **Cortical Volume** | | | **Cortical Surface Area** | | | **Cortical Thickness** | | |
| --- | --- | --- | --- | --- | --- | --- | --- | --- | --- |
|  | **Serum CRP*MDD** | | | **Serum CRP* MDD** | | | **Serum CRP* MDD** | | |
|  | **β** | **SE** | **pFDR** | **β** | **SE** | **pFDR** | **β** | **SE** | **pFDR** |
| **Bank Superior Temporal Sulcus** | 0.060 | 0.058 | 0.910 | 0.041 | 0.058 | 0.935 | 0.055 | 0.061 | 0.966 |
| **Caudal Anterior Cingulate** | -0.045 | 0.052 | 0.910 | 0.009 | 0.053 | 0.935 | -0.025 | 0.061 | 0.966 |
| **Caudal Middle Frontal** | 0.034 | 0.059 | 0.921 | 0.056 | 0.058 | 0.860 | -0.047 | 0.062 | 0.966 |
| **Cuneus** | -0.034 | 0.059 | 0.921 | -0.057 | 0.058 | 0.860 | -0.003 | 0.062 | 0.971 |
| **Entorhinal Cortex** | -0.012 | 0.055 | 0.970 | 0.021 | 0.057 | 0.935 | -0.042 | 0.064 | 0.966 |
| **Frontal Pole** | -0.074 | 0.058 | 0.910 | -0.058 | 0.055 | 0.860 | 0.022 | 0.061 | 0.966 |
| **Fusiform** | 0.035 | 0.054 | 0.921 | 0.074 | 0.054 | 0.860 | -0.027 | 0.066 | 0.966 |
| **Inferior Parietal** | 0.049 | 0.054 | 0.910 | 0.022 | 0.054 | 0.935 | 0.064 | 0.059 | 0.966 |
| **Inferior Temporal** | 0.077 | 0.052 | 0.910 | 0.121 | 0.053 | 0.786 | -0.032 | 0.061 | 0.966 |
| **Insula** | 0.060 | 0.051 | 0.910 | 0.011 | 0.051 | 0.935 | 0.047 | 0.061 | 0.966 |
| **Isthmus Cingulate** | 0.067 | 0.058 | 0.910 | 0.085 | 0.054 | 0.860 | 0.021 | 0.061 | 0.966 |
| **Lateral Occipital** | 0.002 | 0.054 | 0.972 | -0.030 | 0.054 | 0.935 | 0.067 | 0.062 | 0.966 |
| **Lateral Orbito Frontal** | 0.032 | 0.052 | 0.921 | 0.051 | 0.055 | 0.860 | -0.030 | 0.067 | 0.966 |
| **Lingual** | 0.056 | 0.060 | 0.910 | 0.077 | 0.060 | 0.860 | 0.091 | 0.065 | 0.966 |
| **Medial Orbito Frontal** | -0.007 | 0.050 | 0.970 | 0.024 | 0.052 | 0.935 | -0.030 | 0.063 | 0.966 |
| **Middle Temporal** | 0.067 | 0.050 | 0.910 | 0.068 | 0.052 | 0.860 | -0.002 | 0.059 | 0.971 |
| **Paracentral** | 0.085 | 0.057 | 0.910 | 0.049 | 0.056 | 0.860 | 0.069 | 0.059 | 0.966 |
| **Parahippocampal** | -0.042 | 0.062 | 0.921 | 0.048 | 0.057 | 0.862 | -0.119 | 0.067 | 0.966 |
| **Pars Opercularis** | -0.020 | 0.057 | 0.970 | -0.023 | 0.057 | 0.935 | -0.039 | 0.058 | 0.966 |
| **Pars Orbitalis** | -0.010 | 0.053 | 0.970 | 0.022 | 0.054 | 0.935 | -0.021 | 0.060 | 0.966 |
| **Pars Traingularis** | -0.008 | 0.057 | 0.970 | 0.012 | 0.059 | 0.935 | -0.020 | 0.061 | 0.966 |
| **Pericalcarine** | -0.069 | 0.064 | 0.910 | -0.059 | 0.064 | 0.860 | -0.041 | 0.065 | 0.966 |
| **Post Central** | -0.025 | 0.056 | 0.970 | 0.016 | 0.055 | 0.935 | -0.005 | 0.058 | 0.971 |
| **Posterior Cingulate** | 0.003 | 0.056 | 0.972 | 0.031 | 0.053 | 0.935 | -0.037 | 0.063 | 0.966 |
| **Precentral** | 0.015 | 0.057 | 0.970 | 0.014 | 0.054 | 0.935 | 0.007 | 0.065 | 0.971 |
| **Precuneus** | -0.004 | 0.054 | 0.972 | 0.005 | 0.054 | 0.945 | -0.005 | 0.062 | 0.971 |
| **Rostral Anterior Cingulate** | -0.106 | 0.054 | 0.910 | -0.034 | 0.054 | 0.935 | -0.082 | 0.062 | 0.966 |
| **Rostral Middle Frontal** | -0.042 | 0.051 | 0.910 | -0.015 | 0.054 | 0.935 | -0.015 | 0.062 | 0.971 |
| **Superior Frontal** | -0.078 | 0.050 | 0.910 | -0.045 | 0.051 | 0.860 | -0.080 | 0.065 | 0.966 |
| **Superior Parietal** | 0.043 | 0.055 | 0.921 | 0.004 | 0.056 | 0.945 | 0.070 | 0.058 | 0.966 |
| **Superior Temporal** | 0.019 | 0.055 | 0.970 | 0.048 | 0.053 | 0.860 | -0.022 | 0.063 | 0.966 |
| **Supramarginal** | 0.072 | 0.054 | 0.910 | 0.062 | 0.053 | 0.860 | -0.005 | 0.059 | 0.971 |
| **Temporal Pole** | -0.028 | 0.061 | 0.970 | 0.065 | 0.054 | 0.860 | -0.143 | 0.064 | 0.898 |
| **Transverse Temporal** | 0.014 | 0.061 | 0.970 | -0.009 | 0.060 | 0.935 | -0.013 | 0.064 | 0.971 |

**Table S11: Serum CRP* MDD interaction results for individual cortical structures (volume, surface area, thickness)**

| **Individual Brain Structure** | **Cortical Volume** | | | **Cortical Surface Area** | | | **Cortical Thickness** | | |
| --- | --- | --- | --- | --- | --- | --- | --- | --- | --- |
|  | **DNAm CRP*MDD** | | | **DNAm CRP*MDD** | | | **DNAm CRP*MDD** | | |
|  | **β** | **SE** | **pFDR** | **β** | **SE** | **pFDR** | **β** | **SE** | **pFDR** |
| **Bank Superior Temporal Sulcus** | 0.025 | 0.074 | 0.856 | 0.066 | 0.074 | 0.934 | -0.070 | 0.079 | 0.801 |
| **Caudal Anterior Cingulate** | -0.060 | 0.066 | 0.768 | -0.035 | 0.068 | 0.934 | 0.038 | 0.078 | 0.854 |
| **Caudal Middle Frontal** | -0.052 | 0.073 | 0.768 | 0.007 | 0.075 | 0.948 | -0.059 | 0.078 | 0.801 |
| **Cuneus** | 0.027 | 0.076 | 0.856 | 0.103 | 0.075 | 0.934 | -0.065 | 0.081 | 0.801 |
| **Entorhinal Cortex** | -0.131 | 0.071 | 0.768 | -0.088 | 0.074 | 0.934 | -0.022 | 0.083 | 0.871 |
| **Frontal Pole** | 0.047 | 0.074 | 0.768 | -0.026 | 0.072 | 0.934 | 0.080 | 0.078 | 0.801 |
| **Fusiform** | -0.046 | 0.070 | 0.768 | -0.018 | 0.071 | 0.934 | 0.024 | 0.084 | 0.871 |
| **Inferior Parietal** | -0.042 | 0.069 | 0.768 | 0.030 | 0.070 | 0.934 | -0.123 | 0.074 | 0.801 |
| **Inferior Temporal** | 0.088 | 0.067 | 0.768 | 0.060 | 0.069 | 0.934 | 0.065 | 0.077 | 0.801 |
| **Insula** | -0.101 | 0.063 | 0.768 | -0.045 | 0.065 | 0.934 | -0.061 | 0.078 | 0.801 |
| **Isthmus Cingulate** | -0.026 | 0.072 | 0.856 | 0.011 | 0.068 | 0.934 | -0.021 | 0.080 | 0.871 |
| **Lateral Occipital** | 0.052 | 0.069 | 0.768 | 0.069 | 0.071 | 0.934 | -0.032 | 0.078 | 0.871 |
| **Lateral Orbito Frontal** | -0.087 | 0.066 | 0.768 | -0.005 | 0.072 | 0.948 | -0.070 | 0.085 | 0.801 |
| **Lingual** | -0.066 | 0.077 | 0.768 | -0.040 | 0.078 | 0.934 | -0.052 | 0.083 | 0.818 |
| **Medial Orbito Frontal** | -0.146 | 0.064 | 0.768 | -0.072 | 0.066 | 0.934 | -0.058 | 0.081 | 0.801 |
| **Middle Temporal** | 0.046 | 0.063 | 0.768 | 0.062 | 0.067 | 0.934 | -0.038 | 0.075 | 0.854 |
| **Paracentral** | 0.044 | 0.072 | 0.768 | 0.111 | 0.071 | 0.934 | -0.066 | 0.076 | 0.801 |
| **Parahippocampal** | -0.103 | 0.079 | 0.768 | 0.014 | 0.075 | 0.934 | -0.103 | 0.087 | 0.801 |
| **Pars Opercularis** | -0.024 | 0.073 | 0.856 | 0.016 | 0.074 | 0.934 | -0.077 | 0.075 | 0.801 |
| **Pars Orbitalis** | -0.032 | 0.066 | 0.851 | -0.029 | 0.069 | 0.934 | -0.045 | 0.077 | 0.822 |
| **Pars Traingularis** | 0.001 | 0.072 | 0.985 | 0.054 | 0.077 | 0.934 | -0.088 | 0.079 | 0.801 |
| **Pericalcarine** | -0.082 | 0.082 | 0.768 | -0.082 | 0.083 | 0.934 | -0.081 | 0.085 | 0.801 |
| **Post Central** | -0.060 | 0.072 | 0.768 | 0.034 | 0.071 | 0.934 | -0.051 | 0.075 | 0.801 |
| **Posterior Cingulate** | 0.005 | 0.070 | 0.977 | 0.050 | 0.068 | 0.934 | 0.008 | 0.080 | 0.949 |
| **Precentral** | -0.023 | 0.072 | 0.856 | 0.025 | 0.071 | 0.934 | -0.068 | 0.082 | 0.801 |
| **Precuneus** | 0.046 | 0.068 | 0.768 | 0.138 | 0.069 | 0.934 | -0.091 | 0.078 | 0.801 |
| **Rostral Anterior Cingulate** | -0.055 | 0.069 | 0.768 | -0.040 | 0.071 | 0.934 | 0.031 | 0.080 | 0.871 |
| **Rostral Middle Frontal** | -0.071 | 0.064 | 0.768 | -0.026 | 0.070 | 0.934 | -0.095 | 0.080 | 0.801 |
| **Superior Frontal** | -0.076 | 0.063 | 0.768 | -0.010 | 0.066 | 0.934 | -0.110 | 0.081 | 0.801 |
| **Superior Parietal** | -0.011 | 0.069 | 0.926 | 0.023 | 0.072 | 0.934 | -0.054 | 0.075 | 0.801 |
| **Superior Temporal** | 0.047 | 0.069 | 0.768 | 0.078 | 0.067 | 0.934 | 0.012 | 0.080 | 0.940 |
| **Supramarginal** | 0.046 | 0.067 | 0.768 | 0.064 | 0.068 | 0.934 | -0.059 | 0.075 | 0.801 |
| **Temporal Pole** | 0.054 | 0.077 | 0.768 | -0.012 | 0.071 | 0.934 | 0.025 | 0.081 | 0.871 |
| **Transverse Temporal** | 0.022 | 0.078 | 0.857 | 0.014 | 0.078 | 0.934 | 0.004 | 0.083 | 0.959 |

**Table S12: DNAm CRP* MDD interaction results for individual cortical structures (volume, surface area, thickness)**

| **Subcortical Volume** | **(1) Serum CRP* MDD** | | | **(2) DNAm CRP* MDD** | | |
| --- | --- | --- | --- | --- | --- | --- |
|  | **β** | **SE** | **pFDR** | **β** | **SE** | **pFDR** |
| **Nucleus Accumbens** | 0.001 | 0.057 | 0.981 | -0.015 | 0.074 | 0.907 |
| **Amygdala** | 0.024 | 0.058 | 0.981 | 0.103 | 0.074 | 0.601 |
| **Caudate** | 0.062 | 0.062 | 0.981 | 0.106 | 0.080 | 0.601 |
| **Hippocampus** | 0.038 | 0.059 | 0.981 | -0.036 | 0.077 | 0.907 |
| **Pallidum** | -0.014 | 0.058 | 0.981 | 0.025 | 0.075 | 0.907 |
| **Putamen** | 0.009 | 0.059 | 0.981 | 0.071 | 0.076 | 0.701 |
| **Thalamus** | -0.022 | 0.048 | 0.981 | -0.074 | 0.061 | 0.601 |
| **Ventral Diencephalon** | 0.048 | 0.053 | 0.981 | 0.008 | 0.069 | 0.907 |

**Table S13: MDD interaction effects results for both measures of CRP on subcortical volumes**

|  | **DTI Measure** | **Serum CRP* MDD** | | | **DNAm CRP* MDD** | | |
| --- | --- | --- | --- | --- | --- | --- | --- |
|  |  | **β** | **SE** | **pFDR** | **β** | **SE** | **pFDR** |
| **Mean Diffusivity** | gMD | 0.058 | 0.066 | 0.374 | -0.035 | 0.084 | 0.675 |
|  | Average MD | 0.018 | 0.060 | 0.769 | 0.024 | 0.076 | 0.753 |
|  | Association Fibres | 0.055 | 0.066 | 0.535 | -0.045 | 0.084 | 0.622 |
|  | Commisural Fibres | 0.025 | 0.062 | 0.684 | 0.039 | 0.079 | 0.622 |
|  | Projection Fibres | 0.073 | 0.059 | 0.427 | 0.066 | 0.071 | 0.622 |
|  | Thalamic Radiations | 0.080 | 0.053 | 0.427 | 0.051 | 0.065 | 0.622 |
| **Fractional Anisotropy** | gFA | -0.093 | 0.060 | 0.121 | -0.031 | 0.077 | 0.690 |
|  | Average FA | -0.087 | 0.062 | 0.161 | -0.046 | 0.080 | 0.563 |
|  | Association Fibres | -0.056 | 0.061 | 0.354 | 0.015 | 0.078 | 0.849 |
|  | Commisural Fibres | -0.087 | 0.063 | 0.225 | -0.034 | 0.081 | 0.849 |
|  | Projection Fibres | -0.144 | 0.062 | 0.085 | -0.083 | 0.080 | 0.604 |
|  | Thalamic Radiations | -0.120 | 0.060 | 0.090 | -0.083 | 0.077 | 0.604 |

**Table S14: Global DTI results for MDD interaction effects with both measures of CRP**

|  | **Serum CRP* MDD** | | | | | | **DNAm CRP* MDD** | | | | | |
| --- | --- | --- | --- | --- | --- | --- | --- | --- | --- | --- | --- | --- |
| **White Matter Tract** | **Mean Diffusivity** | | | **Fractional Anisotropy** | | | **Mean Diffusivity** | | | **Fractional Anisotropy** | | |
|  | **β** | **SE** | **pFDR** | **β** | **SE** | **pFDR** | **β** | **SE** | **pFDR** | **β** | **SE** | **pFDR** |
| Anterior Corona Radiata | 0.0479 | 0.0695 | 0.9375 | -0.0724 | 0.0633 | 0.4294 | 0.0551 | 0.0859 | 0.9038 | -0.058 | 0.081 | 0.871 |
| Anterior Limb Internal Capsule | 0.0885 | 0.0657 | 0.8562 | -0.1190 | 0.0669 | 0.2486 | -0.0213 | 0.0795 | 0.9486 | -0.074 | 0.085 | 0.871 |
| Cingulum (cingulate gyrus) | 0.0435 | 0.0582 | 0.9375 | -0.0306 | 0.0645 | 0.7617 | -0.0571 | 0.0747 | 0.9038 | -0.007 | 0.083 | 1.000 |
| Cingulum (hippocampus) | -0.0170 | 0.0486 | 0.9375 | -0.0225 | 0.0558 | 0.7806 | 0.0116 | 0.0605 | 0.9486 | 0.040 | 0.072 | 0.893 |
| Corona Radiata | 0.0785 | 0.0672 | 0.9375 | -0.1216 | 0.0650 | 0.2486 | 0.0659 | 0.0802 | 0.9038 | -0.055 | 0.083 | 0.871 |
| Corticospinal Tract | 0.0100 | 0.0619 | 0.9375 | -0.1152 | 0.0631 | 0.2486 | 0.1228 | 0.0789 | 0.9038 | -0.054 | 0.082 | 0.871 |
| External Capsule | 0.0124 | 0.0627 | 0.9375 | -0.0397 | 0.0645 | 0.6824 | -0.0363 | 0.0799 | 0.9181 | -0.057 | 0.081 | 0.871 |
| Fornix(Cres)/ Stria Terminalis | -0.0146 | 0.0523 | 0.9375 | -0.0134 | 0.0542 | 0.8396 | -0.0164 | 0.0653 | 0.9486 | -0.023 | 0.071 | 0.893 |
| Internal Capsule | 0.0817 | 0.0529 | 0.7387 | -0.1018 | 0.0586 | 0.2486 | 0.0326 | 0.0646 | 0.9181 | -0.061 | 0.077 | 0.871 |
| Inferior- Fronto-Occipital Fasciculus | 0.0012 | 0.0591 | 0.9834 | -0.0067 | 0.0630 | 0.9147 | 0.0004 | 0.0758 | 0.9956 | 0.057 | 0.081 | 0.871 |
| Posterior Corona Radiata | 0.0100 | 0.0616 | 0.9375 | -0.1197 | 0.0657 | 0.2486 | 0.0489 | 0.0726 | 0.9038 | -0.073 | 0.083 | 0.871 |
| Posterior Limb of Internal Capsule | 0.0853 | 0.0505 | 0.7326 | -0.0710 | 0.0574 | 0.4132 | 0.0533 | 0.0632 | 0.9038 | -0.029 | 0.075 | 0.893 |
| Posterior Thalamic Radiation | 0.0618 | 0.0599 | 0.9375 | -0.1214 | 0.0605 | 0.2486 | 0.0786 | 0.0759 | 0.9038 | -0.107 | 0.077 | 0.871 |
| Retrolenticular part of Internal Capsule | 0.0297 | 0.0497 | 0.9375 | -0.0683 | 0.0561 | 0.4132 | 0.0432 | 0.0614 | 0.9038 | -0.055 | 0.074 | 0.871 |
| Superior Corona Radiata | 0.1492 | 0.0650 | 0.2652 | -0.1288 | 0.0684 | 0.2486 | 0.0748 | 0.0749 | 0.9038 | -0.021 | 0.088 | 0.928 |
| Superior Fronto-Occipital Fasciculus | 0.1552 | 0.0649 | 0.2652 | -0.0566 | 0.0630 | 0.5541 | 0.0694 | 0.0794 | 0.9038 | 0.001 | 0.081 | 1.000 |
| Superior Longitudinal Fasciculus | 0.0515 | 0.0609 | 0.9375 | -0.0815 | 0.0665 | 0.4132 | -0.0063 | 0.0761 | 0.9744 | 0.035 | 0.084 | 0.893 |
| Sagittal Striatum | 0.0075 | 0.0590 | 0.9375 | -0.1059 | 0.0604 | 0.2486 | 0.1164 | 0.0737 | 0.9038 | -0.146 | 0.077 | 0.871 |
| Uncinate Fasciculus | 0.0349 | 0.0661 | 0.9375 | -0.0244 | 0.0670 | 0.7806 | 0.0918 | 0.0797 | 0.9038 | -0.103 | 0.083 | 0.871 |
| Body Corpus Callosum | 0.0520 | 0.0663 | 0.9375 | -0.0773 | 0.0698 | 0.4294 | 0.0139 | 0.0844 | 0.9486 | 0.000 | 0.090 | 1.000 |
| Corpus Callosum | 0.0282 | 0.0622 | 0.9375 | -0.0810 | 0.0635 | 0.4132 | 0.0391 | 0.0792 | 0.9181 | -0.031 | 0.082 | 0.893 |
| Fornix (column and body) | 0.0519 | 0.0666 | 0.9375 | -0.0388 | 0.0633 | 0.6824 | -0.0539 | 0.0852 | 0.9038 | 0.099 | 0.081 | 0.871 |
| Genu Corpus Callosum | 0.0238 | 0.0641 | 0.9375 | -0.1050 | 0.0634 | 0.2621 | 0.0282 | 0.0822 | 0.9486 | -0.036 | 0.083 | 0.893 |
| Splenium of Corpus Callosum | -0.0117 | 0.0584 | 0.9375 | -0.0361 | 0.0552 | 0.6824 | 0.0715 | 0.0750 | 0.9038 | -0.065 | 0.070 | 0.871 |

**Table S15: Results for MDD interaction effects with both measures of CRP on individual white matter tracts**

|  | **Structural Phenotype** | **MDD Case Control Status** | | | **Total QIDS** | | |
| --- | --- | --- | --- | --- | --- | --- | --- |
|  |  | **β** | **SE** | **pFDR** | **β** | **SE** | **pFDR** |
| **Global Brain Measures** | Global Total Grey Matter | -0.038 | 0.019 | **0.041** | -0.046 | 0.018 | **0.011** |
|  | Global Cerebral White Matter | -0.025 | 0.024 | 0.302 | -0.018 | 0.023 | 0.429 |
|  | Global Cortical Volume | -0.028 | 0.030 | 0.350 | -0.065 | 0.029 | **0.026** |
|  | Global Cortical Thickness | -0.038 | 0.031 | 0.215 | -0.044 | 0.030 | 0.148 |
|  | Global Cortical Surface Area | 0.018 | 0.032 | 0.560 | -0.026 | 0.031 | 0.404 |
|  | Intracranial Volume | -0.059 | 0.030 | **0.048** | -0.086 | 0.029 | **0.003** |
| **Lobar Volume** | Frontal Lobe Volume | -0.036 | 0.022 | 0.154 | -0.043 | 0.022 | 0.059 |
|  | Temporal Lobe Volume | -0.035 | 0.023 | 0.154 | -0.052 | 0.022 | **0.046** |
|  | Parietal Lobe Volume | -0.036 | 0.023 | 0.154 | -0.049 | 0.023 | 0.056 |
|  | Occipital Lobe Volume | -0.079 | 0.026 | **0.014** | -0.066 | 0.026 | **0.046** |
|  | Cingulate Lobe Volume | -0.034 | 0.028 | 0.224 | -0.040 | 0.027 | 0.147 |
| **Lobar Thickness** | Frontal Lobe Thickness | -0.046 | 0.032 | 0.510 | -0.053 | 0.031 | 0.117 |
|  | Temporal Lobe Thickness | -0.031 | 0.033 | 0.510 | -0.040 | 0.032 | 0.210 |
|  | Parietal Lobe Thickness | -0.024 | 0.029 | 0.510 | -0.060 | 0.028 | 0.117 |
|  | Occipital Lobe Thickness | -0.034 | 0.032 | 0.510 | -0.055 | 0.032 | 0.117 |
|  | Cingulate Lobe Thickness | -0.011 | 0.035 | 0.750 | -0.064 | 0.034 | 0.117 |
| **Lobar Surface Area** | Frontal Lobe Surface Area | -0.022 | 0.024 | 0.539 | -0.026 | 0.023 | 0.459 |
|  | Temporal Lobe Surface Area | -0.018 | 0.024 | 0.539 | -0.034 | 0.023 | 0.370 |
|  | Parietal Lobe Surface Area | -0.027 | 0.025 | 0.539 | -0.022 | 0.024 | 0.463 |
|  | Occipital Lobe Surface Area | -0.082 | 0.028 | **0.016** | -0.053 | 0.027 | 0.259 |
|  | Cingulate Lobe Surface Area | -0.017 | 0.027 | 0.539 | 0.000 | 0.026 | 0.988 |

**Table S16: Global and Lobar associations with MDD case/control status and Total QIDS score**

| **Individual Brain Structure** | **Cortical Volume** | | | **Cortical Surface Area** | | | **Cortical Thickness** | | |
| --- | --- | --- | --- | --- | --- | --- | --- | --- | --- |
|  | **MDD Case/Control** | | | **MDD Case/Control** | | | **MDD Case/Control** | | |
|  | **β** | **SE** | **pFDR** | **β** | **SE** | **pFDR** | **β** | **SE** | **pFDR** |
| **Bank Superior Temporal Sulcus** | -0.031 | 0.029 | 0.576 | 0.000 | 0.029 | 0.868 | -0.028 | 0.030 | 0.816 |
| **Caudal Anterior Cingulate** | -0.030 | 0.026 | 0.576 | -0.018 | 0.026 | 0.868 | 0.012 | 0.030 | 0.863 |
| **Caudal Middle Frontal** | -0.008 | 0.029 | 0.851 | -0.008 | 0.029 | 0.868 | -0.038 | 0.031 | 0.755 |
| **Cuneus** | -0.062 | 0.029 | 0.220 | -0.049 | 0.029 | 0.508 | -0.054 | 0.031 | 0.755 |
| **Entorhinal Cortex** | 0.026 | 0.027 | 0.579 | 0.024 | 0.029 | 0.868 | 0.021 | 0.032 | 0.853 |
| **Frontal Pole** | -0.042 | 0.029 | 0.404 | -0.016 | 0.028 | 0.868 | -0.035 | 0.030 | 0.773 |
| **Fusiform** | -0.041 | 0.027 | 0.404 | -0.012 | 0.027 | 0.868 | -0.060 | 0.033 | 0.755 |
| **Inferior Parietal** | -0.001 | 0.027 | 0.966 | -0.001 | 0.027 | 0.966 | 0.000 | 0.029 | 0.989 |
| **Inferior Temporal** | -0.011 | 0.026 | 0.777 | -0.009 | 0.027 | 0.868 | -0.020 | 0.030 | 0.853 |
| **Insula** | -0.025 | 0.025 | 0.576 | -0.020 | 0.026 | 0.868 | -0.033 | 0.031 | 0.799 |
| **Isthmus Cingulate** | 0.002 | 0.029 | 0.966 | 0.012 | 0.027 | 0.868 | -0.011 | 0.030 | 0.863 |
| **Lateral Occipital** | -0.068 | 0.027 | 0.204 | -0.071 | 0.027 | 0.313 | -0.030 | 0.031 | 0.799 |
| **Lateral Orbito Frontal** | 0.016 | 0.026 | 0.679 | 0.020 | 0.028 | 0.868 | -0.017 | 0.033 | 0.853 |
| **Lingual** | -0.061 | 0.030 | 0.220 | -0.070 | 0.030 | 0.342 | -0.009 | 0.032 | 0.863 |
| **Medial Orbito Frontal** | -0.040 | 0.025 | 0.404 | -0.018 | 0.026 | 0.868 | -0.045 | 0.031 | 0.755 |
| **Middle Temporal** | -0.053 | 0.025 | 0.220 | -0.040 | 0.026 | 0.534 | -0.036 | 0.029 | 0.755 |
| **Paracentral** | -0.029 | 0.028 | 0.576 | -0.008 | 0.028 | 0.868 | -0.018 | 0.030 | 0.853 |
| **Parahippocampal** | 0.024 | 0.030 | 0.579 | 0.021 | 0.028 | 0.868 | 0.019 | 0.033 | 0.853 |
| **Pars Opercularis** | 0.002 | 0.028 | 0.966 | -0.005 | 0.029 | 0.897 | -0.024 | 0.029 | 0.853 |
| **Pars Orbitalis** | -0.023 | 0.026 | 0.579 | -0.040 | 0.027 | 0.534 | 0.029 | 0.030 | 0.799 |
| **Pars Traingularis** | -0.064 | 0.028 | 0.220 | -0.053 | 0.029 | 0.498 | 0.010 | 0.030 | 0.863 |
| **Pericalcarine** | -0.048 | 0.032 | 0.404 | -0.058 | 0.032 | 0.498 | -0.016 | 0.032 | 0.853 |
| **Post Central** | -0.042 | 0.028 | 0.404 | -0.028 | 0.027 | 0.852 | -0.008 | 0.029 | 0.863 |
| **Posterior Cingulate** | -0.036 | 0.028 | 0.514 | -0.028 | 0.027 | 0.852 | -0.002 | 0.031 | 0.986 |
| **Precentral** | -0.024 | 0.028 | 0.579 | 0.007 | 0.027 | 0.868 | -0.045 | 0.032 | 0.755 |
| **Precuneus** | -0.021 | 0.027 | 0.579 | 0.006 | 0.027 | 0.868 | -0.043 | 0.030 | 0.755 |
| **Rostral Anterior Cingulate** | -0.014 | 0.027 | 0.737 | 0.010 | 0.027 | 0.868 | -0.041 | 0.031 | 0.755 |
| **Rostral Middle Frontal** | -0.068 | 0.025 | 0.204 | -0.057 | 0.027 | 0.373 | -0.045 | 0.031 | 0.755 |
| **Superior Frontal** | -0.025 | 0.025 | 0.576 | -0.010 | 0.026 | 0.868 | -0.045 | 0.032 | 0.755 |
| **Superior Parietal** | -0.042 | 0.027 | 0.404 | -0.042 | 0.028 | 0.534 | -0.023 | 0.029 | 0.853 |
| **Superior Temporal** | -0.022 | 0.027 | 0.579 | -0.014 | 0.026 | 0.868 | -0.018 | 0.031 | 0.853 |
| **Supramarginal** | -0.020 | 0.027 | 0.579 | -0.022 | 0.027 | 0.868 | -0.008 | 0.029 | 0.863 |
| **Temporal Pole** | 0.010 | 0.030 | 0.832 | 0.012 | 0.027 | 0.868 | 0.016 | 0.032 | 0.853 |
| **Transverse Temporal** | -0.030 | 0.030 | 0.576 | -0.035 | 0.030 | 0.844 | -0.003 | 0.032 | 0.973 |

**Table S17: Results for MDD Case/Control associations with individual cortical structures (volume, surface area, thickness)**

| **Individual Brain Structure** | **Cortical Volume** | | | **Cortical Surface Area** | | | **Cortical Thickness** | | |
| --- | --- | --- | --- | --- | --- | --- | --- | --- | --- |
|  | **Total QIDS** | | | **Total QIDS** | | | **TotalQIDS** | | |
|  | **β** | **SE** | **pFDR** | **β** | **SE** | **pFDR** | **β** | **SE** | **pFDR** |
| **Bank Superior Temporal Sulcus** | -0.036 | 0.028 | 0.302 | -0.015 | 0.028 | 0.799 | -0.012 | 0.029 | 0.757 |
| **Caudal Anterior Cingulate** | -0.032 | 0.025 | 0.302 | -0.012 | 0.026 | 0.817 | -0.032 | 0.029 | 0.458 |
| **Caudal Middle Frontal** | -0.007 | 0.028 | 0.880 | -0.017 | 0.028 | 0.768 | -0.034 | 0.030 | 0.458 |
| **Cuneus** | -0.070 | 0.029 | 0.069 | -0.058 | 0.028 | 0.341 | -0.075 | 0.030 | 0.169 |
| **Entorhinal Cortex** | -0.037 | 0.027 | 0.302 | -0.030 | 0.028 | 0.562 | -0.010 | 0.031 | 0.795 |
| **Frontal Pole** | 0.000 | 0.028 | 0.998 | 0.011 | 0.027 | 0.817 | -0.019 | 0.030 | 0.603 |
| **Fusiform** | -0.034 | 0.026 | 0.302 | 0.009 | 0.026 | 0.817 | -0.071 | 0.032 | 0.194 |
| **Inferior Parietal** | -0.059 | 0.026 | 0.112 | -0.033 | 0.026 | 0.562 | -0.037 | 0.028 | 0.458 |
| **Inferior Temporal** | -0.043 | 0.025 | 0.174 | -0.047 | 0.026 | 0.377 | -0.019 | 0.029 | 0.603 |
| **Insula** | -0.062 | 0.025 | 0.069 | -0.026 | 0.025 | 0.562 | -0.073 | 0.030 | 0.169 |
| **Isthmus Cingulate** | -0.003 | 0.028 | 0.941 | 0.006 | 0.026 | 0.871 | -0.028 | 0.029 | 0.486 |
| **Lateral Occipital** | -0.054 | 0.026 | 0.142 | -0.047 | 0.026 | 0.377 | -0.033 | 0.030 | 0.458 |
| **Lateral Orbito Frontal** | -0.015 | 0.025 | 0.630 | -0.003 | 0.027 | 0.919 | -0.046 | 0.032 | 0.442 |
| **Lingual** | -0.026 | 0.029 | 0.487 | -0.010 | 0.029 | 0.817 | -0.031 | 0.031 | 0.475 |
| **Medial Orbito Frontal** | -0.045 | 0.024 | 0.167 | -0.018 | 0.025 | 0.700 | -0.052 | 0.030 | 0.342 |
| **Middle Temporal** | -0.073 | 0.024 | 0.069 | -0.061 | 0.025 | 0.184 | -0.020 | 0.029 | 0.587 |
| **Paracentral** | -0.046 | 0.027 | 0.174 | -0.010 | 0.027 | 0.817 | -0.063 | 0.029 | 0.194 |
| **Parahippocampal** | 0.024 | 0.030 | 0.538 | 0.032 | 0.028 | 0.562 | -0.009 | 0.032 | 0.811 |
| **Pars Opercularis** | 0.016 | 0.028 | 0.630 | 0.026 | 0.028 | 0.625 | -0.046 | 0.028 | 0.345 |
| **Pars Orbitalis** | -0.043 | 0.026 | 0.174 | -0.038 | 0.027 | 0.562 | -0.035 | 0.029 | 0.458 |
| **Pars Traingularis** | -0.051 | 0.027 | 0.167 | -0.036 | 0.029 | 0.562 | -0.029 | 0.029 | 0.475 |
| **Pericalcarine** | -0.079 | 0.031 | 0.069 | -0.090 | 0.031 | 0.136 | -0.022 | 0.031 | 0.587 |
| **Post Central** | -0.070 | 0.027 | 0.069 | -0.048 | 0.026 | 0.377 | -0.032 | 0.028 | 0.458 |
| **Posterior Cingulate** | -0.020 | 0.027 | 0.565 | 0.011 | 0.026 | 0.817 | -0.032 | 0.030 | 0.473 |
| **Precentral** | -0.075 | 0.027 | 0.069 | -0.040 | 0.026 | 0.555 | -0.057 | 0.031 | 0.310 |
| **Precuneus** | -0.029 | 0.026 | 0.386 | 0.024 | 0.026 | 0.625 | -0.079 | 0.029 | 0.169 |
| **Rostral Anterior Cingulate** | -0.053 | 0.026 | 0.142 | -0.019 | 0.026 | 0.700 | -0.060 | 0.030 | 0.245 |
| **Rostral Middle Frontal** | -0.061 | 0.025 | 0.069 | -0.069 | 0.026 | 0.142 | -0.025 | 0.030 | 0.550 |
| **Superior Frontal** | -0.042 | 0.024 | 0.174 | -0.026 | 0.025 | 0.562 | -0.056 | 0.031 | 0.310 |
| **Superior Parietal** | -0.053 | 0.027 | 0.142 | -0.036 | 0.027 | 0.562 | -0.045 | 0.028 | 0.345 |
| **Superior Temporal** | -0.048 | 0.026 | 0.167 | -0.030 | 0.026 | 0.562 | -0.037 | 0.031 | 0.458 |
| **Supramarginal** | -0.034 | 0.026 | 0.302 | -0.020 | 0.026 | 0.700 | -0.039 | 0.028 | 0.456 |
| **Temporal Pole** | -0.003 | 0.029 | 0.941 | -0.003 | 0.026 | 0.919 | -0.023 | 0.031 | 0.587 |
| **Transverse Temporal** | -0.027 | 0.030 | 0.487 | -0.034 | 0.029 | 0.562 | -0.005 | 0.031 | 0.876 |

**Table S18: Results for Total QIDS associations with individual cortical structures (volume, surface area, thickness)**

| **Subcortical Volume** | **(1) MDD Case/Control** | | | **(2) Total QIDS** | | |
| --- | --- | --- | --- | --- | --- | --- |
|  | **β** | **SE** | **pFDR** | **β** | **SE** | **pFDR** |
| **Nucleus Accumbens** | 0.005 | 0.028 | 0.859 | -0.004 | 0.028 | 0.888 |
| **Amygdala** | -0.018 | 0.029 | 0.732 | 0.032 | 0.028 | 0.729 |
| **Caudate** | 0.019 | 0.031 | 0.732 | -0.006 | 0.030 | 0.888 |
| **Hippocampus** | -0.065 | 0.029 | 0.224 | -0.067 | 0.029 | 0.152 |
| **Pallidum** | -0.019 | 0.029 | 0.732 | 0.004 | 0.028 | 0.888 |
| **Putamen** | 0.037 | 0.030 | 0.569 | -0.011 | 0.029 | 0.888 |
| **Thalamus** | -0.011 | 0.024 | 0.738 | -0.018 | 0.023 | 0.864 |
| **Ventral Diencephalon** | -0.034 | 0.026 | 0.569 | -0.028 | 0.026 | 0.729 |

**Table S19: Results for MDD and QIDS associations with subcortical volumes**

|  | **DTI Measure** | **MDD Case/Control** | | | **Total QIDS** | | |
| --- | --- | --- | --- | --- | --- | --- | --- |
|  |  | **β** | **SE** | **pFDR** | **β** | **SE** | **pFDR** |
| **Mean Diffusivity** | gMD | 0.041 | 0.033 | 0.208 | 0.018 | 0.032 | 0.571 |
|  | Average MD | 0.015 | 0.030 | 0.623 | 0.042 | 0.029 | 0.148 |
|  | Association Fibres | 0.040 | 0.033 | 0.525 | 0.012 | 0.032 | 0.701 |
|  | Commisural Fibres | 0.031 | 0.031 | 0.525 | 0.054 | 0.030 | 0.278 |
|  | Projection Fibres | 0.022 | 0.029 | 0.525 | 0.035 | 0.028 | 0.437 |
|  | Thalamic Radiations | 0.017 | 0.027 | 0.525 | 0.019 | 0.026 | 0.619 |
| **Fractional Anisotropy** | gFA | -0.051 | 0.030 | 0.093 | -0.076 | 0.029 | **0.009** |
|  | Average FA | -0.050 | 0.031 | 0.103 | -0.094 | 0.030 | **0.002** |
|  | Association Fibres | -0.047 | 0.030 | 0.246 | -0.056 | 0.030 | 0.066 |
|  | Commisural Fibres | -0.079 | 0.032 | 0.052 | -0.102 | 0.031 | **0.004** |
|  | Projection Fibres | -0.025 | 0.031 | 0.515 | -0.076 | 0.030 | **0.025** |
|  | Thalamic Radiations | -0.019 | 0.030 | 0.515 | -0.053 | 0.029 | 0.066 |

**Table S20: Global DTI associations with MDD status and Total QIDS score**

|  | **MDD Case/Control** | | | | | | **Total QIDS** | | | | | |
| --- | --- | --- | --- | --- | --- | --- | --- | --- | --- | --- | --- | --- |
| **White Matter Tract** | **Mean Diffusivity** | | | **Fractional Anisotropy** | | | **Mean Diffusivity** | | | **Fractional Anisotropy** | | |
|  | **β** | **SE** | **pFDR** | **β** | **SE** | **pFDR** | **β** | **SE** | **pFDR** | **β** | **SE** | **pFDR** |
| Anterior Corona Radiata | -0.0026 | 0.0345 | 0.9391 | -0.0328 | 0.0316 | 0.4879 | 0.0440 | 0.0335 | 0.3499 | -0.041 | 0.031 | 0.225 |
| Anterior Limb Internal Capsule | 0.0561 | 0.0327 | 0.6924 | -0.0618 | 0.0335 | 0.3139 | 0.0232 | 0.0318 | 0.6213 | -0.051 | 0.033 | 0.167 |
| Cingulum (cingulate gyrus) | 0.0107 | 0.0289 | 0.8096 | -0.0462 | 0.0320 | 0.4495 | 0.0033 | 0.0280 | 0.9463 | -0.067 | 0.031 | 0.090 |
| Cingulum (hippocampus) | -0.0205 | 0.0241 | 0.7611 | 0.0009 | 0.0279 | 0.9752 | 0.0199 | 0.0234 | 0.5581 | -0.059 | 0.027 | 0.090 |
| Corona Radiata | 0.0160 | 0.0335 | 0.7611 | -0.0404 | 0.0325 | 0.4879 | 0.0510 | 0.0325 | 0.3499 | -0.059 | 0.032 | 0.131 |
| Corticospinal Tract | 0.0453 | 0.0309 | 0.7611 | 0.0064 | 0.0316 | 0.8832 | 0.0471 | 0.0301 | 0.3499 | -0.100 | 0.031 | **0.009** |
| External Capsule | 0.0556 | 0.0312 | 0.6924 | -0.0339 | 0.0321 | 0.4879 | 0.0797 | 0.0303 | 0.2080 | -0.057 | 0.031 | 0.131 |
| Fornix(Cres)/ Stria Terminalis | -0.0138 | 0.0259 | 0.7611 | -0.0282 | 0.0271 | 0.4879 | -0.0003 | 0.0252 | 0.9920 | -0.044 | 0.026 | 0.148 |
| Internal Capsule | 0.0231 | 0.0263 | 0.7611 | -0.0219 | 0.0292 | 0.6781 | 0.0222 | 0.0256 | 0.5581 | -0.047 | 0.028 | 0.148 |
| Inferior- Fronto-Occipital Fasciculus | 0.0212 | 0.0295 | 0.7611 | -0.0519 | 0.0314 | 0.3954 | 0.0413 | 0.0287 | 0.3499 | -0.036 | 0.031 | 0.289 |
| Posterior Corona Radiata | 0.0186 | 0.0307 | 0.7611 | -0.0231 | 0.0328 | 0.6781 | 0.0391 | 0.0298 | 0.3499 | -0.059 | 0.032 | 0.131 |
| Posterior Limb of Internal Capsule | 0.0123 | 0.0252 | 0.7611 | -0.0069 | 0.0285 | 0.8832 | 0.0216 | 0.0244 | 0.5581 | -0.014 | 0.028 | 0.632 |
| Posterior Thalamic Radiation | 0.0041 | 0.0298 | 0.9302 | -0.0180 | 0.0302 | 0.6961 | 0.0102 | 0.0289 | 0.8707 | -0.050 | 0.029 | 0.148 |
| Retrolenticular part of Internal Capsule | -0.0081 | 0.0247 | 0.8096 | 0.0054 | 0.0280 | 0.8832 | 0.0090 | 0.0240 | 0.8707 | -0.063 | 0.027 | 0.086 |
| Superior Corona Radiata | 0.0360 | 0.0326 | 0.7611 | -0.0390 | 0.0341 | 0.4879 | 0.0558 | 0.0316 | 0.3499 | -0.055 | 0.033 | 0.148 |
| Superior Fronto-Occipital Fasciculus | 0.0616 | 0.0323 | 0.6924 | -0.0583 | 0.0315 | 0.3139 | 0.0631 | 0.0314 | 0.3499 | -0.057 | 0.031 | 0.131 |
| Superior Longitudinal Fasciculus | 0.0209 | 0.0303 | 0.7611 | -0.0179 | 0.0330 | 0.7059 | 0.0314 | 0.0294 | 0.4902 | -0.029 | 0.032 | 0.426 |
| Sagittal Striatum | -0.0190 | 0.0292 | 0.7611 | -0.0197 | 0.0302 | 0.6860 | 0.0041 | 0.0284 | 0.9463 | -0.045 | 0.029 | 0.171 |
| Uncinate Fasciculus | 0.0394 | 0.0328 | 0.7611 | -0.0376 | 0.0332 | 0.4879 | 0.0448 | 0.0319 | 0.3499 | -0.023 | 0.032 | 0.516 |
| Body Corpus Callosum | 0.0380 | 0.0331 | 0.7611 | -0.0844 | 0.0349 | 0.1625 | 0.0608 | 0.0321 | 0.3499 | -0.099 | 0.034 | **0.022** |
| Corpus Callosum | 0.0312 | 0.0310 | 0.7611 | -0.0799 | 0.0319 | 0.1625 | 0.0571 | 0.0301 | 0.3499 | -0.103 | 0.031 | **0.009** |
| Fornix (column and body) | 0.0369 | 0.0330 | 0.7611 | -0.0324 | 0.0315 | 0.4879 | 0.0068 | 0.0321 | 0.9463 | -0.007 | 0.031 | 0.825 |
| Genu Corpus Callosum | 0.0276 | 0.0318 | 0.7611 | -0.0743 | 0.0320 | 0.1625 | 0.0424 | 0.0310 | 0.3499 | -0.081 | 0.031 | **0.044** |
| Splenium of Corpus Callosum | 0.0179 | 0.0291 | 0.7611 | -0.0430 | 0.0275 | 0.4065 | 0.0481 | 0.0282 | 0.3499 | -0.092 | 0.027 | **0.009** |

**Table S21: Results for MDD case/control status and Total QIDS score associations with individual white matter tracts**

**Supplementary Figure 1:** Associations between Case/Control MDD Status and structural brain phenotypes


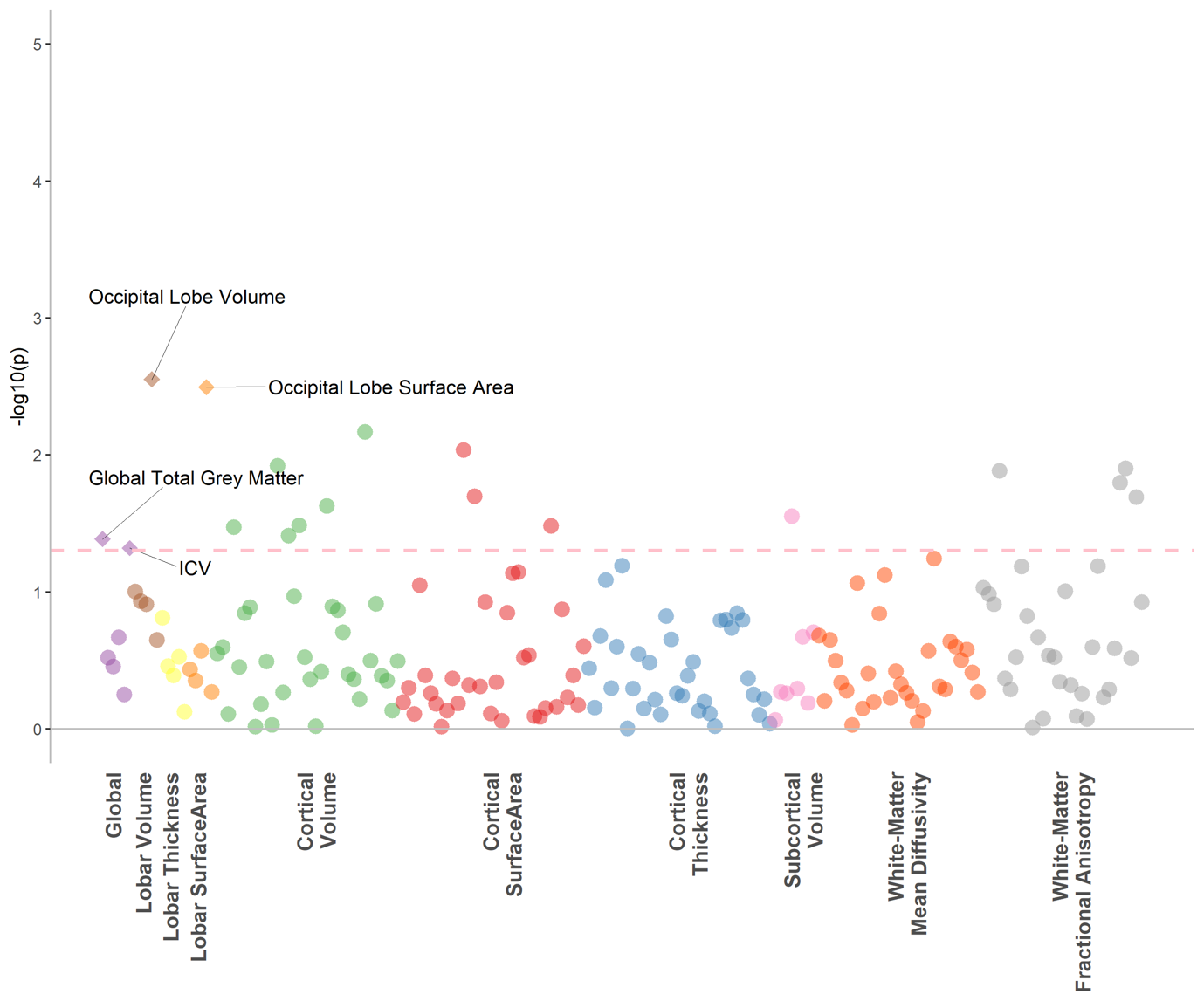


**Figure S1:** The dotted line indicates the p value threshold 0.05. Each dot represents one structural brain phenotype. Each colour represents one imaging modality. The triangular dots represent phenotypes that are also significant after FDR correction.

**Supplementary Figure 2:** Associations between total QIDS score and structural brain phenotypes


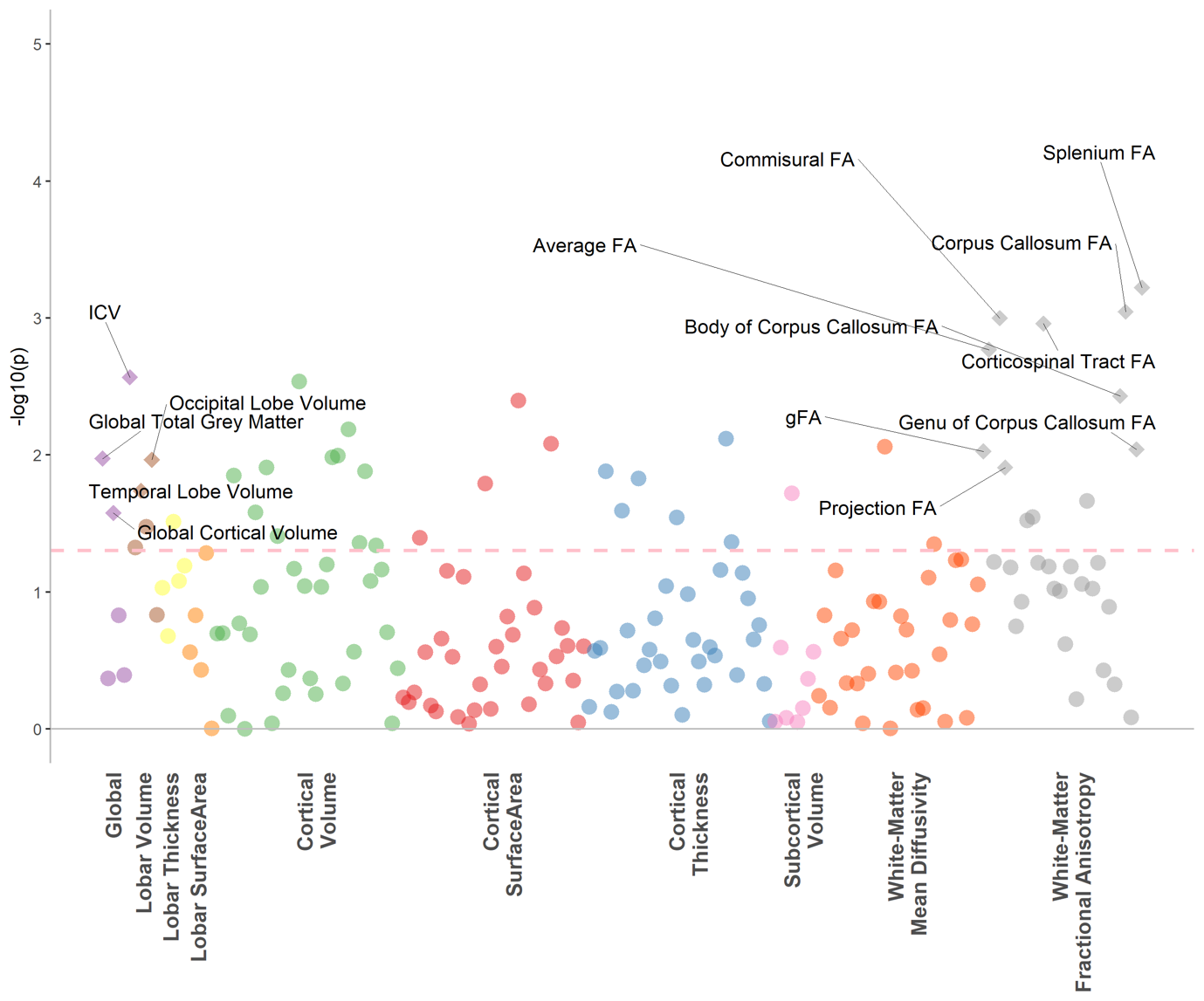


**Figure S2:** The dotted line indicates the p value threshold 0.05. Each dot represents one structural brain phenotype. Each colour represents one imaging modality. The triangular dots represent phenotypes that are also significant after FDR correction.

| **Serum CRP** | | | |
| --- | --- | --- | --- |
| **Measure** | **β** | **SE** | **pFDR** |
| **Total Score** |  |  |  |
| Lifetime MDD | 0.010 | 0.037 | 0.776 |
| Total QIDS | 0.064 | 0.036 | 0.075 |
| **MDD Symptoms** |  |  |  |
| QIDS1- Falling asleep | 0.090 | 0.037 | 0.082 |
| QIDS2- Sleeping during the night | -0.027 | 0.038 | 0.701 |
| QIDS3- Waking up too early | 0.019 | 0.039 | 0.826 |
| QIDS4- Sleeping too much | 0.059 | 0.039 | 0.341 |
| QIDS5- Feeling sad | 0.049 | 0.038 | 0.459 |
| QIDS6- Decreased appetite | 0.040 | 0.045 | 0.611 |
| QIDS7- Increased appetite | -0.009 | 0.051 | 0.923 |
| QIDS8- Decreased weight | -0.017 | 0.051 | 0.844 |
| QIDS9- Increased weight | -0.021 | 0.049 | 0.829 |
| QIDS10- Concentration/decision making | 0.004 | 0.037 | 0.923 |
| QIDS11- View of myself | 0.034 | 0.036 | 0.611 |
| QIDS12- Thoughts of suicide or death | 0.075 | 0.037 | 0.139 |
| QIDS13- General interest | 0.115 | 0.036 | **0.021** |
| QIDS14- Energy level | 0.095 | 0.036 | 0.063 |
| QIDS15- Feeling slowed down | 0.084 | 0.037 | 0.102 |
| QIDS16- Feeling restless | 0.034 | 0.039 | 0.611 |

**Table S22: Supplementary analyses controlling for current smoking status in serum CRP- MDD associations. Covariates= age, sex, BMI, smoking status, site. (n=779)**

| **Clinical Variable** | **(1) Serum CRP** | | | | **(2) DNAm CRP** | | | |
| --- | --- | --- | --- | --- | --- | --- | --- | --- |
|  | **β** | **SE** | **p** | **pFDR** | **β** | **SE** | **p** | **pFDR** |
| Severity Depression (QIDS) | 0.061 | 0.035 | 0.079 | 0.238 | -0.012 | 0.045 | 0.793 | 0.793 |
| Age of Onset (21 or less) | -0.015 | 0.060 | 0.807 | 0.807 | -0.041 | 0.088 | 0.642 | 0.793 |
| Recurrence | 0.022 | 0.061 | 0.723 | 0.807 | -0.071 | 0.089 | 0.428 | 0.793 |

**Table S23: Supplementary Analyses. Associations between serum CRP and DNAm CRP and clinical features of MDD controlling for age, sex, site and BMI. Severity of MDD based on QIDS severity score, Recurrence is defined as more than one episode (n included in analyses=880).**

|  | **Structural Phenotype** | **DNAm CRP** | | |
| --- | --- | --- | --- | --- |
|  |  | **β** | **SE** | **pFDR** |
| **Global Brain Measures** | Global Total Grey Matter | -0.056 | 0.024 | **0.022** |
|  | Global Cerebral White Matter | -0.065 | 0.031 | **0.038** |
|  | Global Cortical Volume | -0.107 | 0.040 | **0.007** |
|  | Global Cortical Thickness | -0.055 | 0.039 | 0.163 |
|  | Global Cortical Surface Area | -0.080 | 0.043 | 0.062 |
| **Lobar Volume** | Frontal Lobe Volume | -0.066 | 0.029 | 0.110 |
|  | Temporal Lobe Volume | -0.055 | 0.030 | 0.116 |
|  | Parietal Lobe Volume | -0.050 | 0.031 | 0.140 |
|  | Occipital Lobe Volume | -0.064 | 0.035 | 0.116 |
|  | Cingulate Lobe Volume | -0.044 | 0.037 | 0.239 |
| **Lobar Thickness** | Frontal Lobe Thickness | -0.052 | 0.042 | 0.400 |
|  | Temporal Lobe Thickness | -0.051 | 0.041 | 0.400 |
|  | Parietal Lobe Thickness | -0.022 | 0.037 | 0.549 |
|  | Occipital Lobe Thickness | -0.041 | 0.041 | 0.400 |
|  | Cingulate Lobe Thickness | -0.048 | 0.046 | 0.400 |
| **Lobar Surface Area** | Frontal Lobe Surface Area | -0.056 | 0.031 | 0.155 |
|  | Temporal Lobe Surface Area | -0.051 | 0.031 | 0.155 |
|  | Parietal Lobe Surface Area | -0.051 | 0.033 | 0.155 |
|  | Occipital Lobe Surface Area | -0.059 | 0.037 | 0.155 |
|  | Cingulate Lobe Surface Area | 0.002 | 0.035 | 0.949 |

**Table S24: Global and Lobar associations with DNAm CRP additionally controlling for time between methylation and imaging/serum collection.**

| **Individual Brain Structure** | **Cortical Volume** | | | **Cortical Surface Area** | | | **Cortical Thickness** | | |
| --- | --- | --- | --- | --- | --- | --- | --- | --- | --- |
|  | **DNAm CRP** | | | **DNAm CRP** | | | **DNAm CRP** | | |
|  | **β** | **SE** | **pFDR** | **β** | **SE** | **pFDR** | **β** | **SE** | **pFDR** |
| **Bank Superior Temporal Sulcus** | -0.058 | 0.037 | 0.402 | -0.060 | 0.037 | 0.416 | -0.066 | 0.039 | 0.267 |
| **Caudal Anterior Cingulate** | -0.033 | 0.033 | 0.544 | -0.013 | 0.034 | 0.799 | -0.014 | 0.039 | 0.932 |
| **Caudal Middle Frontal** | -0.065 | 0.036 | 0.316 | -0.069 | 0.038 | 0.416 | -0.041 | 0.039 | 0.549 |
| **Cuneus** | -0.040 | 0.038 | 0.544 | -0.080 | 0.038 | 0.396 | 0.006 | 0.040 | 0.932 |
| **Entorhinal Cortex** | -0.027 | 0.035 | 0.660 | -0.054 | 0.037 | 0.416 | 0.025 | 0.041 | 0.793 |
| **Frontal Pole** | 0.009 | 0.037 | 0.957 | -0.055 | 0.036 | 0.416 | 0.095 | 0.038 | 0.104 |
| **Fusiform** | -0.088 | 0.035 | 0.136 | -0.056 | 0.035 | 0.416 | -0.088 | 0.042 | 0.170 |
| **Inferior Parietal** | -0.036 | 0.035 | 0.544 | -0.025 | 0.035 | 0.716 | -0.067 | 0.037 | 0.224 |
| **Inferior Temporal** | -0.073 | 0.034 | 0.178 | -0.097 | 0.035 | 0.144 | -0.003 | 0.039 | 0.932 |
| **Insula** | -0.005 | 0.032 | 0.971 | -0.019 | 0.033 | 0.772 | -0.050 | 0.039 | 0.446 |
| **Isthmus Cingulate** | -0.018 | 0.036 | 0.842 | 0.002 | 0.035 | 0.955 | -0.036 | 0.040 | 0.597 |
| **Lateral Occipital** | -0.070 | 0.035 | 0.211 | -0.042 | 0.036 | 0.510 | -0.072 | 0.039 | 0.220 |
| **Lateral Orbito Frontal** | -0.003 | 0.033 | 0.976 | -0.007 | 0.036 | 0.891 | -0.015 | 0.042 | 0.932 |
| **Lingual** | -0.054 | 0.038 | 0.485 | -0.045 | 0.039 | 0.510 | -0.039 | 0.041 | 0.597 |
| **Medial Orbito Frontal** | 0.001 | 0.033 | 0.979 | -0.028 | 0.034 | 0.697 | 0.057 | 0.041 | 0.400 |
| **Middle Temporal** | -0.016 | 0.032 | 0.842 | -0.056 | 0.034 | 0.416 | 0.004 | 0.037 | 0.932 |
| **Paracentral** | -0.035 | 0.036 | 0.544 | -0.031 | 0.036 | 0.688 | -0.053 | 0.038 | 0.400 |
| **Parahippocampal** | -0.047 | 0.040 | 0.544 | 0.018 | 0.038 | 0.785 | -0.105 | 0.043 | 0.104 |
| **Pars Opercularis** | 0.010 | 0.036 | 0.957 | 0.045 | 0.037 | 0.510 | -0.112 | 0.038 | 0.062 |
| **Pars Orbitalis** | -0.080 | 0.033 | 0.136 | -0.062 | 0.035 | 0.416 | -0.010 | 0.038 | 0.932 |
| **Pars Traingularis** | -0.002 | 0.036 | 0.979 | 0.015 | 0.039 | 0.799 | -0.010 | 0.039 | 0.932 |
| **Pericalcarine** | -0.008 | 0.041 | 0.957 | -0.033 | 0.042 | 0.698 | -0.005 | 0.042 | 0.932 |
| **Post Central** | -0.031 | 0.036 | 0.610 | -0.049 | 0.035 | 0.444 | -0.008 | 0.037 | 0.932 |
| **Posterior Cingulate** | -0.048 | 0.035 | 0.485 | -0.016 | 0.034 | 0.785 | -0.036 | 0.040 | 0.597 |
| **Precentral** | -0.113 | 0.036 | 0.065 | -0.051 | 0.035 | 0.416 | -0.120 | 0.041 | 0.062 |
| **Precuneus** | -0.054 | 0.034 | 0.402 | -0.057 | 0.035 | 0.416 | -0.043 | 0.039 | 0.545 |
| **Rostral Anterior Cingulate** | -0.033 | 0.035 | 0.544 | -0.004 | 0.036 | 0.946 | -0.032 | 0.039 | 0.646 |
| **Rostral Middle Frontal** | -0.085 | 0.032 | 0.136 | -0.094 | 0.035 | 0.144 | -0.009 | 0.040 | 0.932 |
| **Superior Frontal** | -0.071 | 0.031 | 0.164 | -0.037 | 0.033 | 0.510 | -0.086 | 0.041 | 0.170 |
| **Superior Parietal** | -0.042 | 0.035 | 0.544 | -0.038 | 0.037 | 0.581 | -0.047 | 0.037 | 0.446 |
| **Superior Temporal** | -0.035 | 0.035 | 0.544 | 0.010 | 0.034 | 0.843 | -0.075 | 0.040 | 0.220 |
| **Supramarginal** | -0.038 | 0.034 | 0.544 | -0.018 | 0.034 | 0.783 | -0.071 | 0.038 | 0.220 |
| **Temporal Pole** | 0.008 | 0.039 | 0.957 | -0.024 | 0.036 | 0.716 | 0.004 | 0.041 | 0.932 |
| **Transverse Temporal** | -0.014 | 0.039 | 0.951 | 0.027 | 0.039 | 0.716 | -0.109 | 0.041 | 0.095 |

**Table S25: DNAm CRP associations with individual cortical brain structures additionally controlling for methylation time**

| **Subcortical Volume** | **DNAm CRP** | | |
| --- | --- | --- | --- |
|  | **β** | **SE** | **pFDR** |
| **Nucleus Accumbens** | -0.007 | 0.037 | 0.944 |
| **Amygdala** | -0.003 | 0.037 | 0.944 |
| **Caudate** | 0.006 | 0.040 | 0.944 |
| **Hippocampus** | 0.008 | 0.038 | 0.944 |
| **Pallidum** | -0.014 | 0.038 | 0.944 |
| **Putamen** | -0.009 | 0.038 | 0.944 |
| **Thalamus** | -0.015 | 0.031 | 0.944 |
| **Ventral Diencephalon** | -0.025 | 0.035 | 0.944 |

**Table S26: DNAm CRP associations with subcortical volumes additionally controlling for time between methylation appointment and imaging/serum collection**

|  | **DTI Measure** | **DNAm CRP** | | |
| --- | --- | --- | --- | --- |
|  |  | **β** | **SE** | **pFDR** |
| **Mean Diffusivity** | gMD | 0.083 | 0.042 | **0.045** |
|  | Average MD | 0.130 | 0.037 | **0.001** |
|  | Association Fibres | 0.073 | 0.042 | 0.080 |
|  | Commisural Fibres | 0.092 | 0.039 | **0.024** |
|  | Projection Fibres | 0.102 | 0.035 | **0.015** |
|  | Thalamic Radiations | 0.082 | 0.032 | **0.023** |
| **Fractional Anisotropy** | gFA | -0.077 | 0.038 | **0.045** |
|  | Average FA | -0.094 | 0.040 | **0.018** |
|  | Association Fibres | -0.093 | 0.039 | 0.065 |
|  | Commisural Fibres | -0.046 | 0.041 | 0.342 |
|  | Projection Fibres | -0.042 | 0.040 | 0.342 |
|  | Thalamic Radiations | -0.037 | 0.039 | 0.342 |

**Table S27: DNAm CRP associations with global DTI measures additionally controlling for time between methylation appointment and imaging/serum collection**

|  | **DNAm CRP** | | | | | |
| --- | --- | --- | --- | --- | --- | --- |
| **White Matter Tract** | **Mean Diffusivity** | | | **Fractional Anisotropy** | | |
|  | **β** | **SE** | **pFDR** | **β** | **SE** | **pFDR** |
| Anterior Corona Radiata | 0.0735 | 0.0426 | 0.1022 | -0.067 | 0.040 | 0.365 |
| Anterior Limb Internal Capsule | 0.1088 | 0.0392 | **0.0312** | -0.126 | 0.042 | **0.034** |
| Cingulum (cingulate gyrus) | 0.0862 | 0.0370 | **0.0349** | -0.058 | 0.041 | 0.429 |
| Cingulum (hippocampus) | 0.0207 | 0.0300 | 0.4906 | -0.032 | 0.036 | 0.514 |
| Corona Radiata | 0.0980 | 0.0397 | **0.0329** | -0.046 | 0.041 | 0.459 |
| Corticospinal Tract | 0.0808 | 0.0391 | 0.0564 | -0.021 | 0.040 | 0.724 |
| External Capsule | 0.1316 | 0.0394 | **0.0109** | -0.137 | 0.040 | **0.016** |
| Fornix(Cres)/ Stria Terminalis | 0.0785 | 0.0323 | **0.0329** | -0.029 | 0.035 | 0.556 |
| Internal Capsule | 0.0770 | 0.0320 | **0.0329** | -0.047 | 0.038 | 0.459 |
| Inferior- Fronto-Occipital Fasciculus | 0.0771 | 0.0374 | 0.0564 | -0.090 | 0.040 | 0.160 |
| Posterior Corona Radiata | 0.1237 | 0.0359 | **0.0109** | -0.018 | 0.042 | 0.724 |
| Posterior Limb of Internal Cpasule | 0.0222 | 0.0314 | 0.4906 | 0.017 | 0.037 | 0.724 |
| Posterior Thalamic Radiation | 0.0744 | 0.0378 | 0.0661 | -0.016 | 0.038 | 0.724 |
| Retrolenticular part of Internal Capsule | 0.0748 | 0.0304 | **0.0329** | -0.038 | 0.037 | 0.459 |
| Superior Corona Radiata | 0.0918 | 0.0370 | **0.0329** | -0.013 | 0.044 | 0.771 |
| Superior Fronto-Occipital Fasciculus | 0.0846 | 0.0389 | **0.0481** | -0.085 | 0.040 | 0.160 |
| Superior Longitudinal Fasciculus | 0.1001 | 0.0375 | **0.0312** | -0.044 | 0.042 | 0.459 |
| Sagittal Striatum | 0.1018 | 0.0367 | **0.0312** | -0.083 | 0.038 | 0.160 |
| Uncinate Fasciculus | 0.0931 | 0.0395 | **0.0347** | -0.044 | 0.041 | 0.459 |
| Body Corpus Callosum | 0.1114 | 0.0415 | **0.0312** | -0.068 | 0.045 | 0.387 |
| Corpus Callosum | 0.0954 | 0.0391 | **0.0329** | -0.053 | 0.041 | 0.459 |
| Fornix (column and body) | 0.0630 | 0.0423 | 0.1492 | -0.065 | 0.040 | 0.365 |
| Genu Corpus Callosum | 0.0768 | 0.0407 | 0.0754 | -0.016 | 0.041 | 0.724 |
| Splenium of Corpus Callosum | 0.0616 | 0.0371 | 0.1110 | -0.036 | 0.035 | 0.459 |

**Table S28: DNAm CRP association with individual white matter tracts additionally controlling for time between methylation appointment and imaging/serum collection.**
